# Psychological advocacy towards healing (PATH): A randomized controlled trial of a psychological intervention in a domestic violence service setting

**DOI:** 10.1371/journal.pone.0205485

**Published:** 2018-11-27

**Authors:** Giulia Ferrari, Gene Feder, Roxane Agnew-Davies, Jayne E. Bailey, Sandra Hollinghurst, Louise Howard, Emma Howarth, Lynnmarie Sardinha, Debbie Sharp, Tim J. Peters

**Affiliations:** 1 Centre for Academic Primary Care, Bristol Medical School, University of Bristol, Bristol, United Kingdom; 2 Department of Global Health and Development, Faculty of Public Health and Policy, London School of Hygiene & Tropical Medicine, London United Kingdom; 3 Domestic Violence Training Ltd, Surrey, United Kingdom; 4 Section of Women’s Mental Health, PO31 King’s College London, London, United Kingdom; 5 NIHR CLAHRC East of England, Douglas House, Cambridge, United Kingdom; 6 School for Policy Studies, University of Bristol, Bristol, United Kingdom; DESPR/NICHD/NIH, UNITED STATES

## Abstract

**Background:**

Experience of domestic violence and abuse (DVA) is associated with mental illness. Advocacy has little effect on mental health outcomes of female DVA survivors and there is uncertainty about the effectiveness of psychological interventions for this population.

**Objective:**

To test effectiveness of a psychological intervention delivered by advocates to DVA survivors.

**Design, masking, setting, participants:**

Pragmatic parallel group individually randomized controlled trial of normal DVA advocacy vs. advocacy + psychological intervention. Statistician and researchers blinded to group assignment. Setting: specialist DVA agencies; two UK cities. Participants: Women aged 16 years and older accessing DVA services.

**Intervention:**

Eight specialist psychological advocacy (SPA) sessions with two follow up sessions.

**Measurements:**

Primary outcomes at 12 months: depression symptoms (PHQ-9) and psychological distress (CORE-OM). Primary analysis: intention to treat linear (logistic) regression model for continuous (binary) outcomes.

**Results:**

263 women recruited (78 in shelter/refuge, 185 in community), 2 withdrew (1 community, control group; 1 intervention, refuge group), 1 was excluded from the study for protocol violation (community, control group), 130 in intervention and 130 in control groups. Recruitment ended June 2013. 12-month follow up: 64%. At 12-month follow up greater improvement in mental health of women in the intervention group. Difference in average CORE-OM score between intervention and control groups: -3.3 points (95% CI -5.5 to -1.2). Difference in average PHQ-9 score between intervention and control group: -2.2 (95% CI -4.1 to -0.3). At 12 months, 35% of the intervention group and 55% of the control group were above the CORE-OM -2clinical threshold (OR 0.32, 95% CI 0.16 to 0.64); 29% of the intervention group and 46% of the control group were above the PHQ-9 clinical threshold (OR 0.41, 95% CI 0.21 to 0.81),

**Limitations:**

64% retention at 12 months

**Conclusions:**

An eight-session psychological intervention delivered by DVA advocates produced clinically relevant improvement in mental health outcomes compared with normal advocacy care.

**Trial registration:**

ISRCTN registry ISRCTN58561170

Original Research

3675/3750

## Introduction

Domestic violence and abuse (DVA) is a common violation of human rights that damages physical and mental health. DVA can be physical, sexual, psychological and economic, perpetrated by a partner, ex-partner or adult family member. Intimate partner violence (IPV) is a type of DVA. Although DVA is experienced by women and men, the majority of severe, repeated and sexual assaults are on women [1]. Most DVA epidemiological research has focused on the impact of intimate partner violence (IPV), a major contributor to the global burden of disease for women of reproductive age [2]. The main long term association of IPV is mental illness, with a three-fold risk of depressive disorders, four-fold risk of anxiety disorders and a seven-fold risk of post-traumatic stress disorder (PTSD) [3]. A meta-analysis of longitudinal studies has established a causal relationship between IPV and depression and suicide attempts [4].

Psychological interventions, such as counselling and cognitive behavioural therapy (CBT) that are not adapted to the specific needs of DVA survivors often fail to meet their needs [5]. Psychological interventions may not directly address the violence, and couple or family therapy, in which victim and perpetrator are treated together, are potentially dangerous. Survivors of DVA have found it unhelpful when interventions do not recognise trauma, make the abuser invisible by focusing exclusively on the mental health of the victim, (implicitly or explicitly) blame the victim for the abuse or her reaction, offer medication rather than counselling and when a psychiatric diagnosis negatively impacts on care or child contact proceedings [6]. In contrast, women identify that interventions can be helpful when they are directly asked about their experiences of DVA, encouraged to name the abuse, helped with safety planning or parenting and offered support to recover from their experiences [7].

There is uncertainty about the effectiveness of psychological interventions designed for survivors of DVA delivered by counsellors or psychotherapists. The systematic review [8] underpinning the 2014 UK National Institute for Health and Care Excellence (NICE) guidelines [9] found two randomized controlled trials of brief psychological interventions for IPV survivors in a refuge and for pregnant women respectively. The former [10], CBT-based, reported improvement in PTSD symptoms; the latter [11], psychotherapy-based, did not. The NICE review also reported four other randomized controlled trials testing cognitive processing, CBT and group therapy, reporting improvement in PTSD and depressive symptoms, but only one study compared the intervention group with a control group with no psychological intervention. Two trials by Kubany and colleagues [12, 13] not included in that review reported reduced post-traumatic stress symptoms following an individual cognitive therapy based intervention in DVA survivors who were no longer in abusive relationships. However, the results from those studies cannot be extrapolated to women suffering from other mental health conditions nor to women still subject to abuse. The World Health Organisation intimate partner violence and sexual violence guidelines [14] recommend trials with sufficient statistical power to assess the effectiveness of different models of psychological intervention/therapy for women survivors of intimate partner violence in a variety of settings. The NICE DVA guidelines [9] recommend research on the effectiveness of psychological interventions modified for domestic violence and abuse in the short, medium and long term, across various levels of risk and including diverse and marginalised groups and programmes for those who have suffered multiple forms of abuse and those who are still experiencing it. In the United States, with the advent of universal screening for IPV in health care settings, there is a renewed emphasis on effective interventions for women disclosing abuse.

In the United Kingdom, domestic violence advocacy or support is provided by a network of specialist DVA services, most affiliated to the Women’s Aid Federation (http://www.womensaid.org.uk/). Advocates engage with individual clients who are being or have been abused, aiming to increase their safety, empower them and link them to community services. The core activities of advocacy are provision of legal, housing and financial advice, facilitating access to and use of community resources such as refuges or shelters, emergency housing, provision of safety planning advice, and ongoing support. The duration and intensity of advocacy varies within and between agencies. Generally advocates do not have a background or training in psychological therapies and do not provide counselling or other therapies. While advocacy may reduce recurrence of violence, its effect on mental health or quality of life is uncertain [15].

Given the contact that advocates have with women who have recently experienced DVA and their understanding of the context of abuse, they are a potential source of psychological support to survivors who seek help from the DVA agencies. Agnew-Davies and colleagues developed a specific intervention for this population delivered by DVA advocates given additional training. The Psychological Advocacy Towards Healing (PATH) intervention is based on concepts and technical strategies drawn from cognitive-behavioural, experiential, dynamic, psycho-educational and feminist theories [16]. This model was evaluated in a before-and-after pilot with a sample of 106 women within refuge (shelter) settings, showing a reduction in participants’ psychological distress [17].

In this paper we report a trial of the PATH intervention in DVA service settings for women in refuges or community-based programmes, testing its clinical effectiveness in terms of mental health outcomes.

## Methods

### Ethics and governance

The PATH trial was approved by the South West 4 (Southmead) Research Ethics Committee, part of the National Research Ethics Service, and site-specific approvals were received from that committee and the South Wales Research Ethics Committee. The conduct of the trial was overseen by an independent Trial Steering Committee (TSC), and an independent Data Monitoring and Ethics Committee (DMEC) that reviewed safety and outcome data throughout the trial. Trial Registration: ISRCTN58561170 (http://www.isrctn.com/ISRCTN58561170). The application for trial registration was submitted to ISRCTN registry on 23.02.2011. Registration was delayed because ISRCTN suggested a fee waiver for National Institute for Health Research funded studies that are adopted into the NIHR portfolio. The adoption process was unexpectedly lengthy and delayed the finalisation of ISRCTN registration, confirmed on 26 July 2011.

### Study design and participants

This is an open, pragmatic or effectiveness (conducted in “real world” circumstances), two parallel group individually randomized controlled trial. The research team, including the statisticians who analysed the outcomes (GFer and TJP), were blind to group assignment. Group assignment was kept in a randomisation database, separate from the analysis database. The randomisation database was only accessible to the trial data manager. The trial manager generated and shared proxy participant IDs and group labels for analysis, and only revealed IDs and random assignment following completion of the analysis, during a meeting between researchers and DVA agencies when results were first announced. Women seeking help from specialist DVA agencies were randomized with a 1:1 ratio to usual DVA agency support (advocacy alone) or usual DVA agency support plus a psychological intervention from an advocate or support worker with additional training: a specialist psychological advocate (SPA). Participants were enrolled in the study for one year, during which time they had access to usual support from the DVA agency. Eligible participants were women aged 16 years or older experiencing DVA which had led them to seek help from one of the recruiting sites. Exclusion criteria: having a psychotic illness, severe drug or alcohol problem; being unable to read English; currently attending counselling, cognitive behaviour therapy or other psychological treatments.

Recruitment took place at two DVA agencies in England and Wales, respectively: Bristol Next Link (http://www.nextlinkhousing.co.uk/) and Cardiff Women’s Aid (http://www.cardiffwomensaid.org.uk/). All women who presented to these agencies were assessed for eligibility by the intake worker and invited to consider participation. A woman potentially interested in participating was referred to a researcher who met with her at a safe location. Informed consent was obtained from the woman in writing if she agreed to participate. After consenting her to the study, the researcher and participant agreed an individualised contact sheet with safety information and details of locators: friends, family or associates who would be able to help the researcher contact the woman. The participant was then asked to complete a booklet of baseline questions. On completion, the participant was randomized to either receiving the PATH intervention or to the control group through a remote independent automated telephone randomisation service provided by the Bristol Randomised Trials Collaboration (http://www.bristol.ac.uk/social-community-medicine/centres/brtc/). Randomisation was stratified by urban area and whether women received support from the refuge/safe house or community based teams in the agencies. Allocation was concealed from the researcher until the moment of randomisation.

Over the course of the study women were asked to self-complete three further questionnaire booklets four, eight and twelve months post-randomisation. Questionnaires were either hand-delivered or sent in the post. Participants were given shopping vouchers for questionnaire completion. Researchers made contact with participants at two, six and ten months post-randomization. Women were reminded either by text, telephone, or post to return questionnaires at two, four and six weeks after questionnaire postal dispatch or hand-delivery. If researchers failed to contact a woman on four consecutive occasions, or the questionnaire was not returned by week seven, the researcher used the women’s locator contacts. Researchers continued to prompt women (or their locators) for the return of questionnaire until week twelve, after which the questionnaire was classified as missing. We issued the next questionnaire in the series even when the previous questionnaire(s) was not returned. Returned questionnaires were checked for completeness and entered into the database.

### Intervention and comparator

#### Specialist psychological advocacy (SPA)

Specialist psychological advocates (SPAs) received a 25-day manualised training programme (available from authors) developed by Agnew-Davies. The training addressed the psychological impacts of DVA on women and developed therapeutic skills specifically tailored for this client group. SPAs were trained to work with common presenting problems within a single session model [18], using a session structure based on the work of Daldrup and colleagues [19]. Topics included post-traumatic stress, depression, anxiety, low self-esteem, unresolved anger and managing loss. SPAs were also provided with handouts and self-help resources that could be used with their clients. Supervision was provided by Agnew-Davies, who listened to a sample of recorded SPA sessions and provided feedback through regular telephone or email contact and monthly face-to-face meetings. Participants in the intervention arm were assigned a SPA with the aim of receiving eight 1:1 SPA sessions (of one hour duration) that alternated with regular advocacy sessions, meeting either weekly or fortnightly with a further two ‘booster’ sessions, one and three months later. During SPA sessions the advocates used a variety of primarily cognitive behavioural psychological techniques focusing within any one session on a specific presenting problem, such as hyper-arousal, sleep difficulties or parenting problems. The SPA aimed to empower the participant to apply therapeutic strategies such as relaxation, challenging thoughts or goal setting, to promote recovery from each problem. In addition to the SPA sessions, women in the intervention group received usual care from their advocate.

#### Usual care

Participants in the advocacy alone group had access to the usual DVA agency support and advocacy, including safety planning, assistance with health and social issues housing problems, budgeting and debt, and legal proceedings. They did not receive SPA sessions and their advocate did not receive specialist training in psychological methods. The length of time a woman was engaged with a DVA agency varied depending on their needs and the service’s policies. The intensity and duration of advocacy support varied.

### Primary outcomes

Clinical Outcomes in Routine Evaluation–Outcome Measure [20] (CORE-OM): 34 questions measuring global psychological distress, with sensitivity to change, good test-retest reliability and UK normative data. It was designed to assess efficacy and effectiveness across multiple disciplines offering psychological therapies [21]. We used it as a continuous measure (mean score) and as a dichotomous measure (clinical cut-off score ≥ 9.9) [22].

Patient Health Questionnaire [23](PHQ-9): nine question measuring symptoms of depression, with sensitivity to change and extensive validation in diverse populations. We used it as a continuous measure (mean score) and as a dichotomous measure (clinical cut-off score ≥ 10 consistent with major depression) [24]. The decision on 11 March 2011 to promote this measure from a secondary to a primary outcome was taken on the recommendation of our TSC and DMEC, and was implemented before the first patient was recruited.

### Secondary outcomes

Generalized Anxiety Disorder questionnaire [25] (GAD7): seven questions measuring symptoms of anxiety, with sensitivity to change, and a score ≥ 10 consistent with a clinical diagnosis of generalised anxiety disorder [26].

PTSD Symptom Scale (PSS) [27] 17 questions measuring post-traumatic stress symptoms with a score ≥17 consistent with a clinical diagnosis of PTSD [28].

Short form-12 [29] (SF-12): 12 item acute form quality of life measure with physical and mental health subscales. Higher SF-12 scores correspond to better health states.

Composite Abuse Scale [30](CAS): 30 questions measuring recent emotional, physical, and severe abuse, as well as harassment, with good sensitivity to change and robust psychometric properties, with increased use in DVA trials [31–33]. We used the CAS to measure both IPV and non-IPV DVA. For this analysis, if a participant was exposed to both types of DVA, we used the larger item score in calculating the total CAS score at each time point.

All primary and secondary outcomes were measured at 4, 8 and 12 months. Data from the PHQ-9 and CORE-OM at 4 and 8 months were treated as secondary outcomes and used for imputation in the absence of the relevant 12 month outcome, because they are clearly correlated with it. We assessed how much contact participants in both intervention and control groups had with their advocate (SPA or non-SPA).

### Serious adverse events

This information was collected and recorded by self-report from the participant, either verbally or in the questionnaire booklet, or elicited by the DVA advocate or SPA following contact with the participant. All serious adverse events were reported to the data monitoring and ethics committee (protocol available from authors).

### Sample size

An effect size of 0.4 to 0.5 is consistent with those detected in studies of psychological interventions using the CORE-OM as an outcome measure [34] and is consistent with findings for CBT interventions on PHQ-9 and other measures of depression [35]. Two hundred participants gives a power of 96% to detect a difference of 0.5 on the CORE-OM (a “reliable change index” [36]), corresponding to an effect size of 0.5, and 81% power to detect an effect size of 0.4. Assuming an attrition of 20%, we aimed to recruit 250 women.

### Statistical analysis

As specified in advance for the primary analyses at 12 months, we analysed groups as randomised, conducting linear regression analyses for continuous, and logistic regression analyses for binary outcomes. These models were used for primary and secondary outcomes at 12 months from randomisation (labelled intention-to-treat, or ITT, here). We adjusted for site (Bristol or Cardiff) and setting (safe house/shelter or community). We did not need to adjust for imbalance in baseline scores of the characteristics of the groups. We adjusted for baseline values of the outcome variables to increase the precision of our estimates. We conducted our pre-specified subgroup analyses by site, setting and age [37], by introducing the relevant interaction effect in our regressions models [38]. As specified in our protocol [37], we investigated whether including participants lost to follow up alters our estimates of effectiveness in sensitivity analyses based on multiple imputation by chained equation models (mice) for the main model [39]. We generated 100 imputed datasets using a mice model with all outcome variables at all time-points, in addition to the variables included in the model for the main analysis (treatment arm plus stratification by urban area and setting). We did not include socio-demographic variables in the mice model, because they did not predict missingness for individual variables. We calculated complier-average causal effects (CACE) using instrumental variables regression techniques to explore a causal link between assignment to treatment and impact in relation to treatment adherence (number of treatment sessions received) [40], using a minimum of four sessions. This analysis was specified in our protocol [37]. It supersedes the generalised mixed model with number of sessions as a fixed categorical effect specified in the protocol as a secondary analysis to assess stability of treatment effect [37], because an instrumental variables-based CACE is better placed to both address and account for the issue of treatment non-adherence [41] (see S1 Text for further details).

We assessed fidelity of the intervention by listening to a stratified random sample of audio-files. A fidelity scale (available from the authors) was developed to measure adherence of SPAs to the PATH model. The scale was adapted from a revised version of the Cognitive Therapy Scale (CTS-R) [42], a widely used measure of competence in CBT, and was used to rate the content of the audiotapes.

We have published details of the trial design [37]. The cost-effectiveness analysis will be reported in a separate paper.

### Funding and conflict of interest

This paper presents independent research funded by the United Kingdom National Institute for Health Research (NIHR) under its Programme Grants for Applied Research scheme (RP-PG-0108-10084). The views expressed in this publication are those of the authors and not necessarily those of the National Health Service, the NIHR, or the Department of Health. The funder had no involvement in study design, conduct or analysis. If the PATH intervention was implemented in service settings, Agnew-Davies would receive payment for training and supervising SPAs. The other authors declare no conflicts of interest.

## Results

We report on the analysis of the primary and secondary outcomes at the primary follow up point of 12 months, between the two groups of women as randomised (ITT).

### Trial flow and baseline characteristics

**Fig 1:**
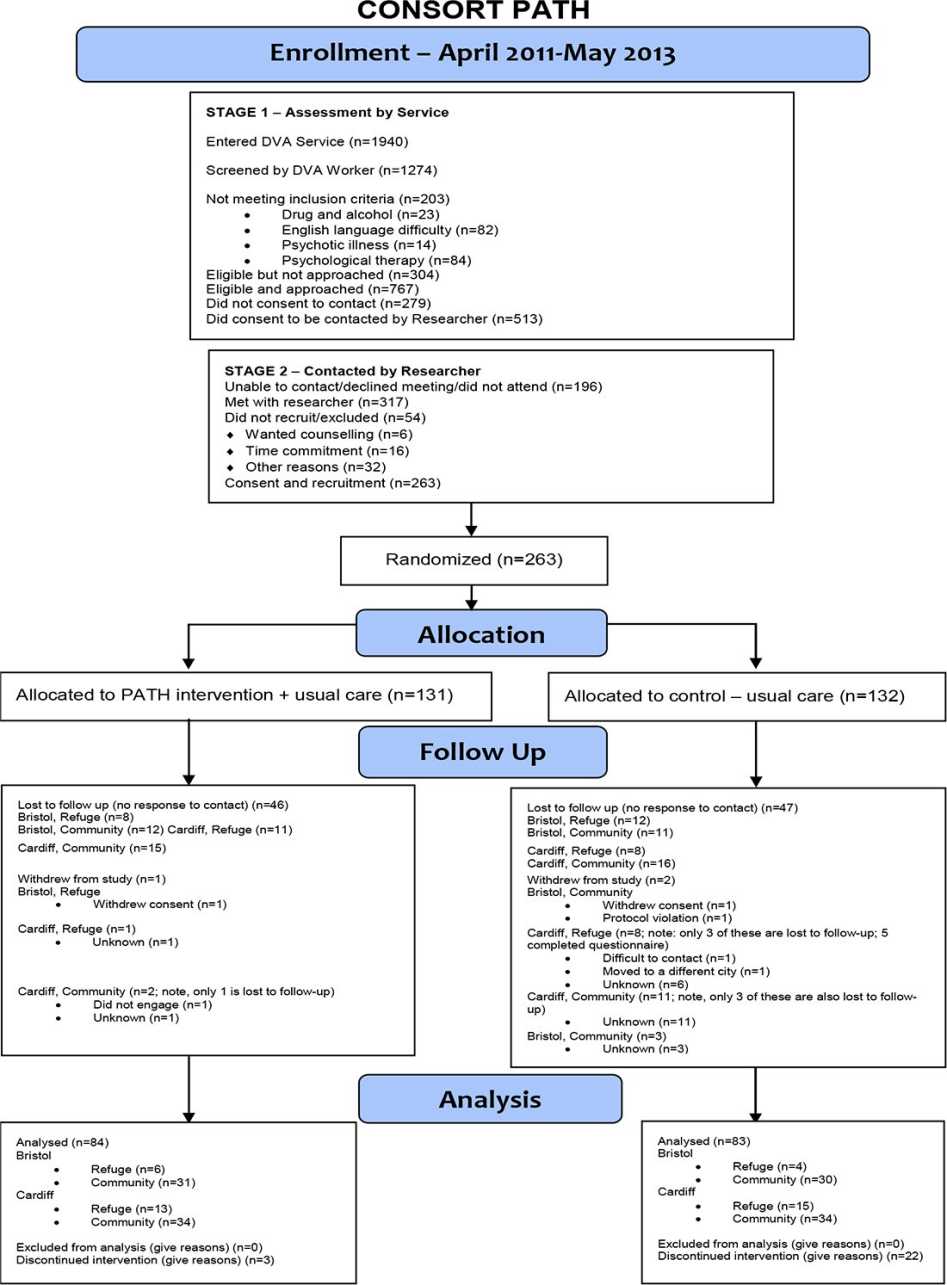
CONSORT diagram illustrating participants‘ flow through the various stages of the trial data collection process.

Between April 2011 and May 2013 we obtained consent and randomized 263 participants: 24% of eligible women seeking help from the two participating DVA agencies (1096), 51% of women who consented to be contacted by a researcher (513), and 83% of women who met with a researcher to discuss participation (317). 64% of participants were retained for the full 12 month follow up period to the end of the trial, although we had primary outcome data from any follow up time point available for 78% of participants (78% SPA group, 77% advocacy alone group). In some cases, it was impossible to establish the reason for loss to follow up, as women had also lost contact with the collaborating agencies (Fig 1). The vulnerability and mobility of women experiencing DVA makes follow up in longitudinal studies challenging [43, 44].

Women lost to follow up were more likely to have been in refuges: 42% lost to follow up, versus 23% lost to follow up among women who were not in a refuge. This would be expected, because women who leave their home for a refuge are more likely to subsequently leave the local area. However, all other socio-demographic characteristics and outcomes did not differ between women lost to follow up and women who remained in the study (S1 Table). In addition, in terms of socio-demographic characteristics or outcomes, women lost to follow up in the control group (N = 43) were comparable to women lost to follow up in the intervention group (N = 47).

The SPA group and advocacy alone group had similar characteristics at baseline (Tables 1 and 2, with more details available elsewhere[45]).

**Table 1:** Baseline characteristics of participants–socio-demographics, alcohol and substance misuse, childhood and abuse.

	Treatment Group			Control Group		
Characteristics	Respondents	Mean (SD) Range (min, max)	Proportion of respondents (%)	Respondents	Mean (SD) Range (min, max)	Proportion of respondents (%)
Age	123	33 (11) (18, 67)		126	34 (10) (18, 65)	
White British or other white background			106/126 (84)			113/127 (89)
Who completed secondary education			96/116 (83)			97/117 (83)
Whose yearly income is at least $17,710*			16/69 (23)			28/87 (32)
Hazardous drinking (Audit-C> = 3)			70/126 (56)			65/125 (52)
Smoked cannabis in past 12 months			36/124 (29)			28/121 (23)
Made use of type A drugs^&^			11/127 (9)			9/125 (7)
Currently in a relationship			26/127 (20)			25/123 (20)
Is parent			97/125 (78)			109/129 (84)
Has child under 4 years living with her			49/130 (38)			47/130 (36)
Perpetrator is a current partner			29/118 (25)			26/118 (22)
Work in the household			46/116 (40)			43/121 (36)
Not in formal employment (excl retirees and students)			90/115 (78)			93/121 (77)
Witnessed DVA as a child			66/129 (51)			67/128 (52)
Abused as a child			65/129 (50)			64/128 (50)

*GBP to USD conversion rate, Nov 01 2012 (source: http://www.oanda.com/currency/converter/)

^&^Heroin (diamorphine), cocaine (including crack), methadone, ecstasy (MDMA), LSD, and magic mushrooms

**Table 2:** Baseline characteristics of participants–domestic abuse and mental health.

	SPA			Control		
Characteristics	Respondents	Mean (SD) Range (min, max)	Proportion of respondents (%)	Respondents	Mean (SD) Range (min, max)	Proportion of respondents (%)
**Composite Abuse Scale (CAS)**						
Total abuse (total score CAS, continuous)	129	59 (35) (0, 138)		129	58 (35) (0, 150)	
Total abuse (total score CAS> = 3)			124/129 (96)			124/129 (96)
Severe abuse (severity CAS> = 1)			89/129 (69)			94/129 (73)
Emotional abuse (emo CAS> = 3)			122/129 (95)			123/129 (95)
Physical abuse (physical CAS > = 1)			119/129 (92)			117/129 (91)
Harassment (harassment CAS> = 2)			113/129 (88)			109/129 (84)
Abused by a family member (not intimate partner)			41/125 (33)			42/127 (33)
Abused for more than 5 years (includes IPV and other domestic abuse)			28/117 (24)			41/121 (34)
Past year experience of abuse (includes IPV and other domestic abuse)			105/117 (90)			112/120 (93)
CORE-OM clinical	130	18 (8) (1, 35)		129	18 (7) (2, 35)	
PHQ-9	130	15 (8) (0, 27)		128	14 (7) (0, 27)	
GAD7	129	13 (6) (0, 21)		126	13 (6) (0, 21)	
PTSD	130	27 (12) (0, 51)		126	26 (11) (0, 51)	
SF12physical	113	48 (12) (21, 68)		123	49 (12) (15, 71)	
SF12mental	113	31 (14) (4, 67)		123	31 (13) (6, 62)	

Note: ‘SPA’ refers to women randomly selected to attend the specialist psychological advocacy treatment. ‘Control’ refers to women randomised to the usual care advocacy group.

Of the 120/130 participants in the SPA group for whom we have information on adherence, 54 (45%) attended fewer than four sessions, and 66 (55%) attended four or more, which we pre-specified as reflecting adequate adherence. Implementation partners also shared records of participants’ attendance at advocacy sessions for all participants. These data show that the average number of advocacy sessions was 5.8 (SD 6.6) for control group participants (N = 124), and 7.2 (SD 6.8) for SPA clients (N = 95).

**Fig 2:**
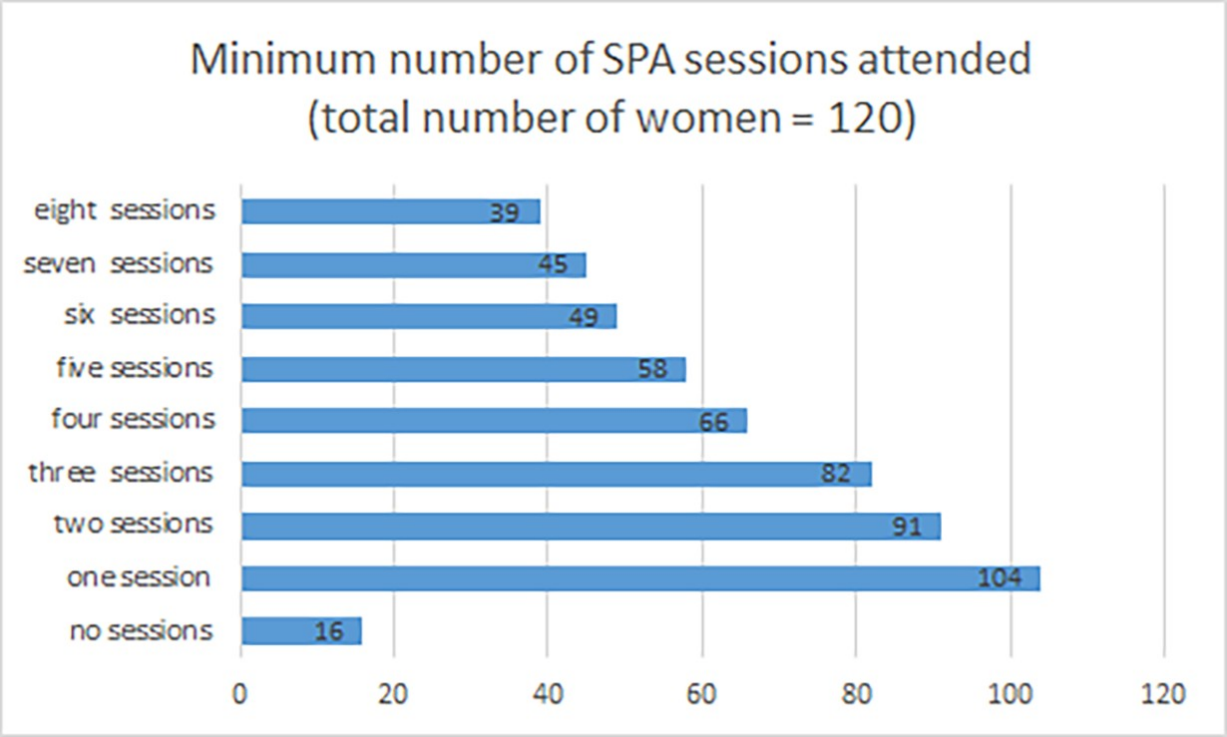
Minimum number of SPA sessions attended. Bars show the number of treated clients attending none, or at least the stated number of sessions.

Fig 2 illustrates the flow of women through the intervention. More than half of the 120 for whom we have attendance records attended four or more of the sessions. One third attended all eight sessions. Thirty-five women also attended the first booster session, and 26 attended both booster sessions.

### Primary outcomes

Table 2 shows a 3.3 point lower mean CORE-OM score and a 2.2 point lower mean PHQ-9 score between the SPA and the advocacy group at 12 months’ follow up. 35% of the SPA group and 55% of the advocacy alone group were above the clinical threshold for the CORE-OM at 12 months respectively (odds ratio 0.32, 95% confidence interval 0.2 to 0.6), with proportions at baseline of 78% and 74% respectively. 29% of the SPA group and 46% of the advocacy alone group were above the clinical threshold for the PHQ-9 at 12 months respectively (odds ratio 0.4, 95% confidence interval 0.2 to 0.8). Trends in the CORE-OM and PHQ-9 score over the 12-month period post randomisation suggest that the intervention group experiences a sharper improvement in mental health four months post recruitment, compared with the control group. Both groups remain stable around the initial reductions over the intervening eight months, suggesting that intervention participants sustain the relative improvement (S1 Fig).

### Secondary outcomes

The PTSD score is lower in the SPA than in the advocacy alone group after 12 months and the proportion of women below the clinical threshold for PTSD is also lower in the SPA group. However, there was no evidence of a difference in GAD-7 score nor the proportion of women below the clinical threshold (Table 3). The SF-12 mental health score is higher in the SPA group, and there is no evidence for a difference in the SF-12 physical health score. There is no evidence of a difference in total exposure to further abuse.

**Table 3:** Differences in primary outcomes at 12 months.

Variable	SPA mean (sd)	Control mean (sd)	Beta (95% CI)		Variable	SPA (n_1_/N_1_)	Control (n_2_/N_2_)	aOR
*Continuous Measures*				*Binary Measures*			
**Primary Outcomes**							
**CORE-OM clinical**	11.3 (8.6)	14.2 (7.9)	-3.3 (-5.5, -1.2)	**CORE-OM caseness**	30/84	46/83	0.32 (0.16, 0.64)
p value			0.003	p value			0.001
N	84	83	166	N			166
**PHQ-9**	7.1 (7.0)	8.9 (6.4)	-2.2 (-4.1, -0.3)	**PHQ-9> = 10**	24/83	38/83	0.41 (0.21, 0.81)
p value			0.021	p value			0.010
N	83	83	165	N			165
**Secondary Outcomes**							
*Measures of Mental Health*							
**PTSD**	15.5 (12.9)	18.9 (12.6)	-3.9 (-7.3, -0.52)	**PTSD> = 17**	36/87	47/83	0.50 (.25, .99)
p value			0.024	p value			0.047
N	87	83	168	N			168
**GAD7**	6.2 (6.1)	7.4 (5.7)	-1.4 (-3.1, 0.36)	**GAD7> = 10**	24/83	24/83	0.95 (0.47, 1.9)
p value			0.12	p value			0.88
N	83	83	163	N			163
*Measures of Health State*							
**SF12** **Mental**	40.5 (15.6)	36.2 (14.0)	4.6 (0.050, 9.2)				
p value			0.048				
N	82	81	150				
**SF12** **Physical**	47.4 (11.0)	48.5 (12.1)	-0.41 (-3.42, 2.6)				
P value			0.79				
N	82	81	150				
*Measure of Abuse (IPV or non-IPV)*							
**Domestic abuse**	16.5 (28.9)	21.9 (29.7)	-5.1 (-13.9, 3.7)	**Abuse cases**	37/81	49/82	0.56 (0.30, 1.05)
p value			0.251	p value			0.071
N			161	N			161

Note: beta is the coefficient on the treatment (SPA) variable in the linear regression models for continuous outcomes; aOR are adjusted odds-ratios from the logistic regressions for binary outcomes. ‘SPA’ refers to women randomly selected to attend the specialist psychological advocacy treatment. ‘Control’ refers to women randomised to the usual care advocacy group. Estimates are adjusted for site (Bristol or Cardiff) and setting (safe house/shelter or community), as well as baseline values of the relevant outcome variable.

### Pre-specified secondary analyses

For primary and secondary outcomes, the multiple imputation for missing data gave lower point estimates for differences between intervention and control groups, although all were within the 95% confidence intervals of the complete case analysis (S2 Table). The imputations for binary outcomes yielded very similar odds ratios to the complete case analysis (S2 Table). The CACE analysis results in roughly a doubling of the effect of the intervention across mental health and abuse variables, consistent with greater benefit for those participants attending a sufficient number of SPA sessions (S2 Table).

The fidelity analysis (details available from the authors) shows that SPAs broadly adhered to the session structure and the working alliance with participants, as specified in the PATH manual and training.

The subgroup analyses found no evidence of interaction effects between treatment and age, site and setting (S3 Table).

### Serious adverse events

22 women (15 in the SPA group, seven in the advocacy alone group) reported a total of 32 serious adverse events (Table 4). For three women (four events) in the intervention group, it was unclear from the information available whether the SAEs were related to trial participation.These events were reported to the NHS research ethic committee (REC) that had approved the study, following advice from the chair of our study’s independent Data Monitoring & Ethics Committee. The REC judged that nochanges to the conduct of the intervention or trial were necessary. None of the remaining 28 SAEs (19 women) were related to trial participation. Most of the serious adverse events were hospital admission due to physical or mental ill health. This included six cases of attempted suicide, seven cases related to pregnancy or miscarriage, three cases of injuries from physical assault, ten cases of chest or abdominal pain and two planned admissions. Planned admissions and injuries from physical assault were only found in the intervention arm. All other non-miscellaneous categories occurred with a similar frequency in both arms.

**Table 4:** Serious adverse events by randomisation arm.

SAE type	Random assignment	
	SPA (N)	Control (N)
**Attempted suicide**	4	2
**Planned**	2	0
**Pregnancy-related**	5	2
**Injuries from physical assault**	3	0
**Chest/abdominal pain**	7	3
**Other**	2	2
**Total**	23	9

Note: ‘SPA’ refers to women randomly selected to attend the specialist psychological advocacy treatment. ‘Control’ refers to women randomised to the usual care advocacy group.

## Conclusions

This study is the first randomized controlled trial of an intervention to improve mental health symptoms of women experiencing DVA delivered by advocates with additional training in psychological methods. The PATH intervention, unlike the majority of psychological treatments for survivors of DVA, which are delivered by psychologists or counsellors, is based on a relatively brief training of advocates already in the DVA sector with ongoing supervision by a psychologist.

We found that the primary outcomes of psychological distress and symptoms of depression were reduced in both arms compared to baseline, with evidence of a difference between the groups at 12 months follow up. This was also the case for post-traumatic stress symptoms and a general measure of mental health state (SF-12mental), but not anxiety or experience of further abuse.

Although the differences in mean scores for the primary outcomes between the groups are relatively modest, there are clinically important differences in the primary outcomes expressed as proportions of participants in the intervention and control groups with depression and with psychological distress. The treatment effect is reduced with imputation for missing data and increased in those participants attending more SPA sessions. A judgement about whether the extent of benefit justifies implementation of the PATH intervention needs to take into account the findings of the nested qualitative study, reported in the accompanying qualitative paper (see Evans et al., DOI: 10.1371/journal.pone.0193077), that explores the experiences of participants.

The four SAEs in which the relation to trial participation was uncertain occurred in the control group. The greater number of SAEs over all recorded in the intervention group was probably due to greater contact with DVA advocates.

### Strengths and limitations

Strengths of PATH include: recruitment of a sizeable proportion of the eligible women experiencing abuse and seeking help [46]; successful collaboration with agencies in the DVA sector to deliver a psychological intervention with reasonable fidelity; relatively complete data collection from participants retained in the trial; outcome data from at least one time point after randomisation from 74% of participants; and a nested longitudinal qualitative study (see Evans et al., DOI: 10.1371/journal.pone.0193077). The main limitation threatening the internal validity of the trial is the 34% loss of participants 12 months post-intervention, requiring pre-specified imputation of the missing outcome data. The main limitation threatening its external validity is the proportion of eligible women who did not participate, including those who wanted counselling and potentially most likely to respond to the intervention. Finally, this was a “real world” pragmatic trial, where intervention participants had higher levels of contact with services because they accessed SPA treatment in addition to usual care. The absence of an attention control group means we cannot exclude the possibility that the additional contact was responsible for the treatment effect. However, our IV results in conjunction with advocates’ adherence to the treatment model (see fidelity analysis) provides support for the hypothesis that the increased effectiveness deriving from attendance may be explained by the treatment, rather than more exposure to advocates.

### Comparison with other studies

The standardised effect size of the SPA intervention (0.4 for CORE-OM and 0.32 for PHQ-9) is similar to the effect of psychological treatment for depression in primary care [47]. The effect on mental health outcomes is comparable to other trials of psychological interventions for survivors of DVA measuring treatment impact on trauma-related mental health symptoms or wellbeing, as systematically reviewed by Warshaw and colleagues [48]. Five of the nine studies in that review were CBT-based interventions for women who were experiencing IPV and three were targeted at women with additional conditions: suicidality, substance abuse or pregnancy. All the interventions were delivered by trained therapists, whereas the PATH intervention was delivered by domestic violence advocates with relatively brief training, but ongoing supervision.

### Implications of findings

The PATH intervention delivered by specialist psychological advocates can improve psychological distress and depressive symptoms in women survivors of DVA, the majority of whom had experienced IPV. The findings of the trial and of the nested qualitative study (see Evans et al., DOI: 10.1371/journal.pone.0193077) show heterogeneity in response to the intervention; further abuse, experienced by a large minority of participants, possibly attenuated its effect. These findings from a pragmatic trial can be implemented in DVA agency settings that already employ advocates, if they undergo further training.

## Supporting information

S1 Table Participants successfully followed up at 12 months vs participants who were not, baseline characteristics. (DOCX)

S2 Table Complete case analysis compared with mice and CACE estimates. (DOCX)

S3 Table Subgroup analyses. (DOCX)

S4 Table CONSORT checklist PATH. (DOC)

S1 Fig Main outcomes, time trends. (DOCX)

S1 Text Methodological discussion of the instrumental variables estimates for PATH. (DOCX)

S2 Text Trial of an individually randomised, parallel group controlled trial to determine if a psychological intervention delivered by domestic violence advocates is effective and cost-effective: Psychological Advocacy Towards Healing (PATH). (DOC)

S3 Text PATH protocol paper (Trials 2013). (PDF)
