# Supplementary material for: Psychological advocacy towards healing (PATH): A randomized controlled trial of a psychological intervention in a domestic violence service setting
Source: PLoS One. 2018 Nov 27;13(11):e0205485. doi: 10.1371/journal.pone.0205485 (PMC6258512; doi:10.1371/journal.pone.0205485)
Supplement: S1 Table — (DOCX) [file pone.0205485.s001.docx]

**S1 Table. Participants successfully followed up at 12 months vs participants who were not, baseline characteristics**

|  | Women who responded at 12 months | | | Women who did not respond at 12 months | | |
| --- | --- | --- | --- | --- | --- | --- |
|  | Respon  dents | Mean (SD) | Proportion of respondents (%) | Respon  dents | Mean (SD) | Proportion of respondents (%) |
| Age | 157 | 33.73 (11.12) |  | 92 | 32.95 (10.55) |  |
| CORE-OM clinical | 165 | 17.88 (7.50) |  | 94 | 18.54 (7.51) |  |
| PHQ9 | 165 | 14.05 (7.35) |  | 93 | 15.25 (7.51) |  |
| GAD7 | 163 | 13.03 (6.00) |  | 92 | 13.10 (6.36) |  |
| PTSD | 164 | 26.33 (12.01) |  | 92 | 26.43 (11.81) |  |
| SF12physical | 152 | 48.81 (11.56) |  | 84 | 47.79 12.87) |  |
| SF12mental | 152 | 31.48 (12.83) |  | 84 | 30.11 14.37) |  |
| Total abuse (total score CAS, continuous) | 164 | 57.63 (34.19) |  | 94 | 60.35 (35.21) |  |
| White ethnicity | . |  | 140/162 (0.86) | . |  | 79/91 (0.87) |
| Who completed secondary education | . |  | 125/147 (0.85) | . |  | 68/86 (0.79) |
| Whose yearly income is at least $17,710  GBP to USD conversion rate, Nov 01 2012 (source: <http://www.oanda.com/currency/converter/>) | . |  | 31/100 (0.31) | . |  | 13/56 (0.23) |
| Hazardous drinking (Audit-C>=3) | . |  | 87/160 (0.54) | . |  | 48/91 (0.53) |
| Smoked cannabis in past 12 months | . |  | 36/158 (0.23) | . |  | 28/87 (0.32) |
| Made use of type A drugs2 in past 12 months  (Heroin (diamorphine), cocaine (including crack), methadone, ecstasy (MDMA), LSD, and magic mushrooms ) | . |  | 13/161 (0.08) | . |  | 7/91 (0.08) |
| Currently in a relationship | . |  | 34/159 (0.21) | . |  | 17/91 (0.19) |
| Is parent | . |  | 135/162 (0.83) | . |  | 71/92 (0.77) |
| Has child under 4 years living with her | . |  | 62/166 (0.37) | . |  | 34/94 (0.36) |
| Perpetrator is a current partner | . |  | 33/149 (0.22) | . |  | 22/87 (0.25) |
| Work in the household | . |  | 59/155 (0.38) | . |  | 30/82 (0.37) |
| Not in formal employment (excl retirees and students) | . |  | 117/154 (0.76) | . |  | 66/82 (0.80) |
| Witnessed DVA as a child | . |  | 82/165 (0.50) | . |  | 51/92 (0.55) |
| Abused as a child | . |  | 78/165 (0.47) | . |  | 51/92 (0.55) |
| Total abuse (total score CAS>=3) | . |  | 157/164 (0.96) | . |  | 91/94 (0.97) |
| Severe abuse (severity CAS>=1) | . |  | 117/164 (0.71) | . |  | 66/94 (0.70) |
| Emotional abuse (emo CAS>=3) | . |  | 154/164 (0.94) | . |  | 91/94 (0.97) |
| Physical abuse (physical CAS >=1) | . |  | 148/164 (0.90) | . |  | 88/94 (0.94) |
| Harassment (harassment CAS>=2) | . |  | 137/164 (0.84) | . |  | 85/94 (0.90) |
| Abused by a family member (not intimate partner) | . |  | 47/158 (0.30) | . |  | 36/94 (0.38) |
| Domestic abuse for more than 5 years (IPV and other) | . |  | 41/150 (0.27) | . |  | 28/88 (0.32) |
| Domestic abuse, past year experience (IPV and other) | . |  | 138/149 (0.93) | . |  | 79/88 (0.90) |
