# Supplementary material for: Psychological advocacy towards healing (PATH): A randomized controlled trial of a psychological intervention in a domestic violence service setting
Source: PLoS One. 2018 Nov 27;13(11):e0205485. doi: 10.1371/journal.pone.0205485 (PMC6258512; doi:10.1371/journal.pone.0205485)
Supplement: S2 Table — (DOCX) [file pone.0205485.s002.docx]

## S2 Table. Complete case analysis compared with mice and CACE estimates

| Outcome | CACE estimates | Complete case | mice estimates |
| --- | --- | --- | --- |
| Primary Outcomes | | | |
| CORE-OM | | | |
| difference between intervention and control group mean scores | -5.98 | -3.3 | -2.8 |
| 95% CI | (-9.8, -2.2) | (-5.5, -1.2) | (-4.9, -0.68) |
| p value | 0.002 | 0.003 | 0.010 |
| N | 160 | 166 | 260 |
|  |  |  |  |
| PHQ9 | | | |
| difference between intervention and control group mean scores | -3.8 | -2.2 | -1.8 |
| 95% CI | (-7.04, -0.46) | (-4.1, -0.3) | (-3.7, 0.088) |
| p value | 0.025 | 0.021 | 0.062 |
| N | 159 | 165 | 260 |
| Secondary Outcomes | | | |
| *Measures of Mental Health* | | | |
| PTSD |  |  |  |
| difference between intervention and control group mean scores | -6.9 | -3.9 | -3.5 |
| 95% CI | (-12.8, -0.96) | (-7.3, -0.52) | (-6.9, -0.042) |
| p value | 0.023 | 0.024 | 0.047 |
| N | 162 | 168 | 260 |
|  |  |  |  |
| GAD7 |  |  |  |
| difference between intervention and control group mean scores | -2.38 | -1.4 | -0.99 |
| 95% CI | (-5.3, 0.50) | (-3.1, 0.4) | (-2.8, 0.77) |
| p value | 0.106 | 0.12 | 0.269 |
| N | 157 | 163 | 260 |
|  |  |  |  |
| *Measures of Health State and Health State Utility* | | | |
| SF-12 Mental Health |  |  |  |
| difference between intervention and control group mean scores | 8.4 | 4.6 | 0.87 |
| 95% CI | (0.44, 16.4) | (0.050, 9.16) | (-1.84, 3.6) |
| p value | 0.039 | 0.048 | 0.53 |
| N | 144 | 150 | 260 |
|  |  |  |  |
| SF-12 Physical Health |  |  |  |
| difference between intervention and control group mean scores | 0.26 | -0.41 | 0.86 |
| 95% CI | (-4.9, 5.4) | (-3.41, 2.6) | (-1.35, 3.07) |
| p value | 0.920 | 0.79 | 0.44 |
| N | 144 | 150 | 260 |
|  |  |  |  |
| *Measure of Abuse* | | | |
|  |  |  |  |
| CAS (either IPV or non-IPV DA) |  |  |  |
| difference between intervention and control group mean scores | -8.4 | -5.1 | -2.7 |
| 95% CI | (-24.3, 7.4) | (-13.9, 3.7) | (-11.7, 6.2) |
| p value | 0.30 | 0.251 | 0.55 |
| N | 155 | 161 | 260 |
