# Supplementary material for: Psychological advocacy towards healing (PATH): A randomized controlled trial of a psychological intervention in a domestic violence service setting
Source: PLoS One. 2018 Nov 27;13(11):e0205485. doi: 10.1371/journal.pone.0205485 (PMC6258512; doi:10.1371/journal.pone.0205485)
Supplement: S3 Table — (DOCX) [file pone.0205485.s003.docx]

## S3 Table Subgroup analyses

| **Variable** | **Intervention group average (sd)** | **Control group average (sd)** | **Difference between intervention and control group mean scores** | **Interaction tests** |
| --- | --- | --- | --- | --- |
| **COREOM clinical** | | | | |
|  | 11.3 (8.6) | 14.2 (7.9) | -3.3 |  |
| 95% CI |  |  | (-5.5, -1.2) |  |
| p value |  |  | 0.003 |  |
| N | 84 | 83 | 166 |  |
| *Subgroup analyses* | | | | |
| **Age binary** | | | | |
|  |  |  |  |  |
| **LR chi2(2)** |  |  |  | 2.11 |
| **Prob >chi2** |  |  |  | 0.347 |
| **Age continuous** | | | | |
| **LR chi2(2)** |  |  |  | 0.48 |
| **Prob >chi2** |  |  |  | 0.7873 |
| **Site** | | | | |
| **LR chi2(2)** |  |  |  | 0.82 |
| **Prob >chi2** |  |  |  | 0.3644 |
| **Service** | | | | |
| **LR chi2(2)** |  |  |  | 0.45 |
| **Prob >chi2** |  |  |  | 0.5026 |
|  |  |  |  |  |
| **PHQ9** | | | | |
|  | 7.1 (7.0) | 8.9 (6.4) | -2.2 |  |
| 95% CI |  |  | (-4.1, -0.34) |  |
| p value |  |  | 0.021 |  |
| N | 83 | 83 | 165 |  |
| *Subgroup analyses* | | | | |
| **Age binary** | | | | |
| **LR chi2(2)** |  |  |  | 1.10 |
| **Prob >chi2** |  |  |  | 0.5765 |
| **Age continuous** | | | | |
| **LR chi2(2)** |  |  |  | 0.70 |
| **Prob >chi2** |  |  |  | 0.7045 |
| **Site** | | | | |
| **LR chi2(2)** |  |  |  | 1.18 |
| **Prob >chi2** |  |  |  | 0.2771 |
| **Service** | | | | |
| **LR chi2(2)** |  |  |  | 0.01 |
| **Prob >chi2** |  |  |  | 0.9238 |
