# Supplementary material for: Psychological advocacy towards healing (PATH): A randomized controlled trial of a psychological intervention in a domestic violence service setting
Source: PLoS One. 2018 Nov 27;13(11):e0205485. doi: 10.1371/journal.pone.0205485 (PMC6258512; doi:10.1371/journal.pone.0205485)
Supplement: S1 Fig — (DOCX) [file pone.0205485.s005.docx]

**S4 Fig. Main outcomes, time trends**

**Panel A. Average CORE-OM score over time, by treatment arm**

**Panel B. Average PHQ9 score over time, by treatment arm**

Average CORE-OM( panel A) and PHQ9 (panel B) scores, by treatment arm, at each time point. Means are weighted by sampling design (city (Bristol/Cardiff) and setting (in the community/refuge))
