# Supplementary material for: Psychological advocacy towards healing (PATH): A randomized controlled trial of a psychological intervention in a domestic violence service setting
Source: PLoS One. 2018 Nov 27;13(11):e0205485. doi: 10.1371/journal.pone.0205485 (PMC6258512; doi:10.1371/journal.pone.0205485)
Supplement: S1 Text — (DOCX) [file pone.0205485.s006.docx]

### S5 Text. Methodological discussion of the instrumental variables estimates for PATH

In a real world setting, some of the women who are offered the treatment will not take it up. We want to capture this possibility with our primary estimates, and therefore compute intention-to-treat estimates over all study participants as randomised, independently of treatment uptake in the intervention group [1].

However, we observe non-adherence to treatment in our intervention group (Figure 1 in the paper). In addition, it is possible that the specific patterns of non-adherence to treatment that we observe in our finite sample may differ from those observed in the population of domestic violence survivors who contact advocacy agencies. Therefore, we also compute the effect of actually receiving treatment in those women who took up treatment (defined as attending at least 4 sessions), given they have been offered access, compared to women who were not treated [2, 3]. This allows us to compute a complier-average causal effect (CACE) within an instrumental variable (IV) framework, where random assignment is our instrument for treatment exposure. The 4-session threshold described in the paper was identified *a priori* by the psychologist who devised the treatment, and divides women in the intervention group in two groups of approximately equal size, with 66 out of 120 respondents having attended at least 4 sessions.

Conditions for the use of IV estimation [1-4] were satisfied as follows.

Random treatment assignment ensures the absence of confounding between treatment assignment and mental health outcomes: random assignment as implemented in this study [5] satisfies the independence assumption, i.e. that treatment assignment is independent of the unmeasured confounding. This statement is further supported by the successful randomisation on observables, including mental health measures, shown in Table 1 in the paper.

The monotonicity assumption, i.e. the assumption that assignment to receive the intervention does not decrease the level of treatment [6], is ensured by design in this trial [3, p.62]. Women in the control group could not access the PATH treatment, so that random assignment directly and positively impacts the likelihood of exposure to treatment. The data confirm that no woman randomised to the control group received the PATH treatment, and that 55% of the women randomised to receiving the intervention complied with treatment, i.e. attended four or more sessions.

The exclusion restriction, i.e. that randomisation has an impact on women’s wellbeing only via attendance, is broadly satisfied. The study was a single-blind randomised trial, where the researchers, and the statisticians in particular, were blind to treatment assignment. However, treatment assignment could not be feasibly concealed from clients and advocates, due to its nature and the real-world conditions in which the treatment was administered. This implies that there is some risk that knowledge of having been assigned to receiving the intervention may have altered the mental health outcomes of treated compared with control women. Similarly, SPAs’ and advocates’ knowledge of clients’ treatment status may have introduced differences in their interactions with intervention and control clients respectively, which may have affected clients’ mental health outcomes. However, we took measures to mitigate this bias in various ways. First, SPA workers only interacted with intervention women, and non-SPA advocates only interacted with control women, continuing their work as usual. No trained SPA treated control women, so as to reduce the likelihood that they behave differently with control as opposed to intervention women. Moreover, the fidelity study revealed that SPA workers adhered to the PATH delivery model, further supporting the hypothesis that the experience of treatment exposure was consistent for clients randomly assigned to treatment. Finally, our participant information leaflet clarified that the efficacy (and effectiveness) of the treatment was unknown, all study participants received the same amount of attention from researchers in terms of follow-up requests and support while filling in the baseline questionnaire, and random assignment was only determined after the baseline questionnaire had been completed. These precautions were designed to minimise the risk that women assigned to treatment would feel more or better looked after than women assigned to the control group.

Overall, these precautions reduced the risk that women in the control group would receive less attentive care than women in the intervention group, and hence minimise the extent to which any treatment effect derived from influences other than receipt of the intervention.

In addition, the first stage estimations report F-statistics of about 30 in all cases, suggesting that the instrument is strong by commonly accepted standards, and that the IV estimate is unlikely to be inflated by a weak instrument [3]. In addition, first stage post-estimation tests suggest that the additional instrument (random assignment) has significant explanatory power for exposure to treatment after controlling for study design. Finally, for all outcomes but anxiety, exogeneity of treatment exposure to treatment impact is rejected at the 95% confidence level, suggesting the appropriateness of IV regression as opposed to standard regression techniques to estimate the effect of receiving the intervention on mental health outcomes.

1. Ten Have TR, Normand S-LT, Marcus SM, Brown CH, Lavori P, Duan N. Intent-to-Treat vs. Non-Intent-to-Treat Analyses under Treatment Non-Adherence in Mental Health Randomized Trials. Psychiat Ann. 2008;38(12):772-83. doi: 10.3928/00485713-20081201-10. PubMed PMID: PMC2921714.

2. Baiocchi M, Cheng J, Small DS. Tutorial in Biostatistics: Instrumental Variable Methods for Causal Inference*. Statistics in medicine. 2014;33(13):2297-340. doi: 10.1002/sim.6128. PubMed PMID: PMC4201653.

3. Hernan MA, Robins JM. Instruments for causal inference: an epidemiologist's dream? Epidemiology (Cambridge, Mass). 2006;17(4):360-72. Epub 2006/06/07. doi: 10.1097/01.ede.0000222409.00878.37. PubMed PMID: 16755261.

4. Greenland S. An introduction to instrumental variables for epidemiologists. International journal of epidemiology. 2000;29(4):722-9.

5. Brierley G, Agnew-Davies R, Bailey J, Evans M, Fackrell M, Ferrari G, et al. Psychological advocacy toward healing (PATH): study protocol for a randomized controlled trial. Trials. 2013;14(1):221. PubMed PMID: doi:10.1186/1745-6215-14-221.

6. Imbens GW, Wooldridge JM. Recent developments in the econometrics of program evaluation. Journal of economic literature. 2009;47(1):5-86.
