# Supplementary material for: Psychological advocacy towards healing (PATH): A randomized controlled trial of a psychological intervention in a domestic violence service setting
Source: PLoS One. 2018 Nov 27;13(11):e0205485. doi: 10.1371/journal.pone.0205485 (PMC6258512; doi:10.1371/journal.pone.0205485)
Supplement: S2 Text — (DOC) [file pone.0205485.s007.doc]

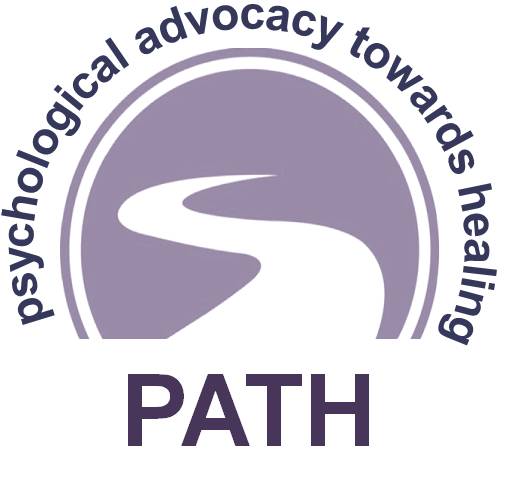


1. Title

Trial of an individually randomised, parallel group controlled trial to determine if a psychological intervention delivered by domestic violence advocates is effective and cost-effective: Psychological Advocacy Towards Healing (PATH)

2. Investigators

Gene Feder (principal investigator)

Debbie Sharp

Tim Peters

Alice Malpass

Maggie Evans

Fayeza Saeed

Sandra Hollinghurst

*University of Bristol*

Roxane Agnew Davies

*Domestic Violence Training Ltd, London*

Carol Metters

*Next Link, Bristol*

Morgan Fackrell

Cardiff Women’s Aid

**3. Aims**

- - 1. To determine whether women experiencing DV and referred or self-referred to a specialist DV agency will have improved quality of life and mental health outcomes if they receive a psychological intervention alongside DV advocacy compared with advocacy alone
    2. To determine whether the psychological intervention is cost-effective.

**4. Objectives**

# To train eight domestic violence advocates, four at Bristol based advocacy organisation Next Link and four from Cardiff Women’s Aid to implement the intervention

4.2.1 To recruit **125** women to the intervention programme and **125** to the control arm

4.2.2 To follow up the women for 12 months from baseline measurements post-recruitment.

4.2.3 To administer baseline and 4 monthly follow up measures for 12 months from recruitment.

**5. Background**

5.1 Prevalence of domestic violence

Domestic violence is threatening behaviour, violence or abuse (psychological, physical, sexual, financial or emotional) between adults who are or have been intimate partners or family members. The 2004/5 British Crime Survey reported that 28% of women had experienced abuse from a partner or ex-partner at some time in their lives, with a 19% prevalence of physical assault and 24% sexual assault.^1^ Although acts of violence against men by women partners are not uncommon, these are generally less severe, with fewer long term consequences and mostly do not occur in a context of coercive control.^2^ Sexual violence against men by women partners is rare. The prevalence of domestic violence among women seeking health care is higher than in the general population. In the first primary care-based study in the UK, we found a lifetime experience of physical and sexual violence of 40%, with 17% of women experiencing domestic violence in the past year.^3^ Almost one third of women interviewed in Scottish primary care reported experience of domestic abuse at some point in their lives; for 45% of these women, the abuse lasted for over five years.^4^ Domestic violence is a major public health problem to which primary care historically has failed to respond adequately.^5^ The cost of domestic violence to the UK economy, including costs to the State, to employers and in human suffering is £23 billion.^6^ The issue of domestic violence is particularly important in the context of general practice, because of the extensive contact between women and primary care clinicians (90% consult over 5 years) and because women themselves identify primary care clinicians as the people from whom they would seek support.^7^

5.2 Mental health consequences

The impact of domestic violence has psychological parallels with the trauma of being taken hostage and subjected to torture.^8^ ^9^ Over and above damage to physical^10^ ^11^ and reproductive health,^12^ domestic violence has long term detrimental effects on mental health. In Golding’s landmark meta-analysis^13^ of studies, mostly from north America, measuring the relationship between domestic violence and mental illness, she reported increased risks for a range of conditions:

OR (95% c.i.)

- depression: 18 studies 3.8 (3.2 to 4.6)
- PTSD: 11 studies 3.74 (2.1 to 6.8)
- alcohol abuse: 9 studies 5.6 (3 to 9)
- suicidality: 13 studies 3.55 (2.7 to 4.6)

Our UK based study of women with a lifetime experience of domestic violence attending general practices in east London^14^ found odds ratios greater than three for depression, anxiety and PTSD and greater than two for suicide attempt, use of illegal drugs and alcohol abuse.

Self-harm has become of increasing concern in the UK particularly in young Asian women, linked to experiences of domestic violence and forced marriage[[1]](#endnote-2). Black and Asian women are at considerably more risk of attempting suicide, with prevalence ratios 1.6 times that of white women and 2.5 times that of Asian men, rising to 2.5 and 7 respectively for young Asian women (under 30, but often married). Domestic violence has been identified in 49% of suicide attempts made by black women compared to 22% of suicide attempts by white women.[[2]](#endnote-3)

Domestic violence may be the single most important cause of female suicidality.

5.3 Psychological interventions

Although there is some evidence of spontaneous improvement in mental health and quality of life of women who have left an abusive partner, mental health problems persist for years.^15^. We have systematically reviewed the evidence for domestic violence advocacy and psychological interventions on quality of life and mental health outcomes.^16^ ^17^. Globally, there are only six controlled studies of individual psychological interventions for women who have experienced domestic violence, *none* in the UK and only three measuring depression outcomes. The six studies demonstrated modest) improvements (effect sizes ranged from 0.25 to 2.33) in psychological outcomes including depression, PTSD and self-esteem. Two randomized controlled trials of individual cognitive therapy based interventions for women with PTSD who were no longer experiencing violence showed significant improvement in PTSD symptoms, but this cannot be extrapolated to women suffering from other mental health conditions nor women still in abusive relationships.

Clinical research with survivors of domestic violence (Herman 1992) distinguishes between stages of recovery for women who have experienced physical or sexual violence. Zimmerman (2006) recommends a three step process: crisis/emergency intervention; support for women’s recuperation and psychological adjustment; and care for long-term symptom management. As such, the initial needs of women at risk of further violation are to gain safety and security. (Foa & Meadows, 1997). Those who are secure can then process emotional and psychological sequelae. However, there is also a distinction between short-term symptoms, which can be resolved with help and longer-term symptoms or vulnerability, which may be much less amenable to change.

5.3 DV-competent therapy

Women with a recent history of domestic violence have mental health needs that are not being met in primary care or mental health services. Standard psychological interventions such as counselling and CBT that are not adapted to the specific needs of this vulnerable group are not effective and potentially dangerous. The NICE 2005 guidelines on the management of depression highlight the need for trauma focused psychotherapy in severe complex cases. We have developed an intervention (the Refuge model) designed and piloted by Agnew-Davies and colleagues with a sample of 106 women within refuge settings^18;19^ funded by the Department of Health (section 64 funding programme). This intervention has not yet been tested with community populations. Using the clinical outcomes in routine evaluation-outcome measure (CORE-OM) measure, they found a reduction of mean total CORE score from 1.72 to 1.34 (clinical cut off = 1.29).

The Refuge model draws from different concepts and technical strategies within cognitive-behavioural, experiential, dynamic, psycho-educational and feminist theories. Theoretical and technical choice affords the offer of alternatives based on the question: ‘What would be the best approach with this (client) with this problem in this setting at this time?’ ^20^ The approach is practical and eclectic, because no single psychotherapy can provide an adequate breadth of interventions ^21^ and is informed by very brief intervention models ^22^

5.4 DV-competent therapy delivered by domestic violence advocates

Within the UK, there is a network of domestic violence advocacy services, most affiliated to the Women’s Aid Federation. Specialist domestic violence advocates provide support to women who are referred from other services or self-referred and help them access resources in the community, including legal advice, housing and financial support. Generally they do not have a background or training in psychological therapies and do not provide counseling or other therapies. In our research programme, we are currently conducting a cluster randomized controlled trial to improve identification of domestic violence survivors in general practice and referral to advocacy services. (IRIS: ISRCTN74012786). Although survivors are well supported by these services, their mental health needs are not being addressed. As discussed above, referral to standard counseling or mental health services is unlikely to be effective and is potentially dangerous, particularly if there is still contact with the perpetrator. With the appropriate training, domestic violence advocates can deliver a specialist trauma-focused intervention to women who are referred from primary care or self referred to their partner domestic violence agencies.

**6. Intervention and control arms**

6.1 Training of advocates in psychological intervention

We will recruit four experienced advocates based at Cardiff Women’s Aid and four from Next Link, a Bristol-wide domestic violence agency that already collaborates in the IRIS trial and is a co-applicant in the NIHR applied research programme grant application. The seconded advocates will receive a 25-day training from Agnew-Davies: five day blocks separated by a one week break after blocks two and four. The first module covers domestic violence awareness focussed on assessing the effects on psychological health and therapeutic principles working with this specialist population. The second module expands risk assessment and safety planning from a psychological perspective and delineates strategies for promoting recovery from Post Traumatic Stress Disorder. Subsequent sessions address psychological problems commonly presented by victims of intimate partner violence, including depression, anxiety, low self-esteem and managing loss. Once implementation starts, the advocates will attend fortnightly individual and monthly group supervision with Agnew Davies.

6.3 Intervention arm

8 hour-long weekly sessions tailored to the needs of the individual woman and 1-2 booster sessions over the next 6 months. The advocates will provide time-limited interventions using a variety of (primarily) cognitive behavioural psychological techniques focussing within any one session on a specific presenting problem, such as hyper-arousal, sleeping difficulties or parenting problems. The advocate will empower the client to apply therapeutic strategies (such as diary keeping, challenging thoughts or goal setting) to promote recovery from each problem. While the intervention is manualised, each package will be tailored to promote needs-led flexibility in the problem focus and the order in which problems are addressed over an eight session contract. Common elements characterising all interventions will include a focus on domestic violence as an aetiological factor, post-traumatic stress as a framework and attention to the therapeutic alliance.

6.4 Control arm

This would be normal support from advocates, varying from a single session and referral to other agencies or regular meetings over a period of months.

**7. Trial design**

7.1 Participants

Women referred or self referred to Next Link or Cardiff Women’s Aid for domestic violence advocacy.

7.1.1 Inclusion criteria

- female
- aged => 16 years

7.1.2 Exclusion criteria

- psychotic illness
- unable to read English
- severe current drug or alcohol abuse
- currently attending counselling, CBT or other psychological treatments either in primary care or specialist psychiatric services

Current or past use of antidepressants or benzodiazepines is *not* an exclusion criterion.

7.1.3 Recruitment

Potential participants will be approached by the advocacy service intake worker at Next Link/Cardiff Women’s Aid who will ask all women meeting the inclusion criteria if they would be willing to speak a researcher from the University of Bristol about the research trial they are collaborating on. The contact details and safety preferences of women who consent to being contacted about the study will be transmitted to the researcher via telephone in the presence of the potential participant and will be kept strictly confidential. When initial telephone contact has been made with a woman, the researcher will arrange a face-to-face meeting with all women interested and eligible to take part, meeting the woman at a safe and convenient place. As part of the meeting, the researcher will give a detailed participant information sheet to the woman, explaining each point and answer any questions that the woman may have prior to seeking her written informed consent. Written consent for interviews and digital audio−recording of SPA sessions will be sought separately in addition from all women who have indicated their willingness to take part in interviews on the initial consent form.

Randomisation will be independent of the researcher and the participant will be informed which arm she is by the researcher. The advocates delivering the intervention will not have contact with the women in the control arm.

7.2 Randomisation

Participants will be randomly allocated, in a1:1 ratio to the intervention and control arms respectively via a computer program accessed remotely by the recruiting researcher. Information about allocation will be communicated to Next Link/Cardiff Women’s Aid by the researcher recruiting. Allocation will be stratified by whether women receive support from the safe house team or resettlement team at Next Link, or the refuge team or tenant support team at Cardiff Women’s Aid.

7.3 Setting

The intervention will be delivered either at premises of Next Link/Cardiff Women’s Aid, where private rooms are available, or at another venue deemed safe and convenient for the researcher and participant including participant’s homes or refuges.

7.4 Quantitative measures

7.4.1 Mental health measures

- CORE-OM: 34-item self-completed measure of psychological distress that provides a mean overall score (range 0–4) and four domain scores (well-being, problems, functioning and risk). This acceptable, standardised, valid and reliable outcome measure, with sensitivity to change, and UK normative data, was designed to assess efficacy and effectiveness across multiple disciplines offering psychological therapies.^24^
- PHQ-9
- GAD-7
- Sheehan’s (1986) Post-Traumatic Stress Symptom Questionnaire

The primary outcome of the trial will be a mental health measure, the CORE-OM, for which we already have pilot data in relation to the intervention. To detect a reduction of 0.4 SD on the CORE with a power of 80% at a 5% significance level would require 250 participants in the trial.

7.4.2 Abuse measures (baseline, 4 month, 8 month and 12 month follow up)

- Composite Abuse Scale (CAS): 30 item self-completed measure of partner abuse with good acceptability and reliability, with some evidence of criterion and external validity. ^25^ Measures partner lifetime and past 12 month partner abuse (can be adapted for more recent abuse)
- Child abuse measure (three questions)

7.4.3 Short Form-12

7.4.4 EQ-5D

7.4.5 Previous mental health problems (self-reported), including treatment

7.4.6 Socio-demographic measures including age, number of children at home, ethnicity, income, occupation and relationship to perpetrator

7.4.7 Cost data

- health service use of participants (self report)
- travel costs
- intervention training and delivery costs

7.4.8 Process measures

- numbers of sessions attended
- digital recording of treatment sessions with independent evaluation of intervention fidelity
- supervision sessions attended by advocates

**8. Analysis**

- Intention-to-treat and per-protocol analyses, including all randomised patients for whom outcome data are available. Analysis of covariance with the one year CORE-OM score as the dependent variable, adjusted for baseline CORE-OM score and the minimisation variables. A standardised effect size will be calculated by dividing the effect size by the SD, pooled across the 2 groups with a sensitivity analysis for the effect of missing data with conservative assumption. To assess the stability of any treatment effect we will fit a mixed model for CORE-OM scores at 4, 8, and 12 months, adjusted for baseline CORE-OM and minimisation variables, using treated number of sessions as a fixed categorical effect. Binary and continuous secondary outcome measures will be analysed with logistic regression and analysis of covariance, respectively, adjusted for baseline CORE-OM scores and minimisation variables.
- With directly collected patient and treatment costs we will calculate cost-effectiveness from NHS and societal perspectives, using EQ-5D scores to generate quality adjusted life years and expressing these findings in terms of incremental cost per QALY gained.

**9. Nested qualitative study**

(i) To explore advocate and client perceptions of the intervention from one-to-one repeated interviews

(ii) inform interpretation of the trial findings as well as dissemination and implementation, if the trial is positive. The repeated interviews with advocates will articulate barriers and facilitators to delivering the intervention.

8.1 Participant observation

- Observations of the process of delivering the intervention will occur throughout the study. Observations will include the training of all advocates and the delivery of the intervention. These observations will be carried out by the trainer and supervisor of advocates (RAD), and the director of Next Link (CM) and Cardiff Women’s Aid (MF), who directly manage the advocates. The main purpose of the observations will be to support validity through triangulation and better understand the processes and assumptions that underlie the potential effectiveness of the intervention. Informed consent for observations will be sought in each new setting and the data recorded in a fieldwork diary.

8.2 Repeat in-depth interviews with participants, advocates and managers

- The aim of interviews with 8 SPA advocates and 4 managers (4 SPA and 2 managers from each site) will be to explore barriers and facilitators to delivering the intervention. Topic guides will include questions that explore what changes had to be made to the intervention (manual guide) in order to tailor it for individual women’s needs; whether women with a longer history of abuse responded differently to the intervention; which parts of the intervention seemed most helpful for women and possible reasons for this.
- The aim of interviews with 50 participants will be to explore women’s assessment of their mental well being and quality of life in their own words, at the beginning and end of the 8 one-hour weekly sessions (i.e. week one and week eight) as well as the overall acceptability and perceived effectiveness of the intervention. Topic guides would include questions that explore which of the therapeutic strategies (such as diary keeping, challenging thoughts or goal setting) were most useful to the women and why. Interviews would be face-to-face and last up to one hour. With participants’ consent, interviews would be recorded and transcribed verbatim. Purposive sampling, based on data collected in a baseline questionnaire will ensure a diverse group of informants, in terms of age, currency of abuse, and mental health status. Interviews will be conducted by ME and AM.

1. **Analysis**

The data will be analysed using Framework,^26^ an approach that is useful when linking analysis to quantitative findings, in this case to individual level outcomes. In Framework analysis all the key issues, concepts, and themes by which the data can be examined and referenced are identified. This is carried out by drawing on a priori issues and questions derived from the aims and objectives of the study as well as issues raised by the respondents themselves and views or experiences that recur in the data. ^27^

**11. User participation**

This proposal has been developed collaboratively with Next Link, a voluntary organisation with a strong user advisory group. GF has presented the design of the pilot and the main trial to 10 members of the advisory group. Their comments and suggestions have informed the final design of the pilot. The service users who attended this meeting have agreed to meet as a reference group for the trial times two times per year, allowing the investigators to report on progress of the study and seek advice on problems. If we are successful in our programme grant application we will also use this group as a reference group for the whole PROVIDE programme.

Reference List

(1) Finney A. Domestic violence, sexual assault and stalking: Findings from the 2004/05 British Crime Survey. 12/06 online report. London: Home Office; 2006:

(2) AuCoin K (editor). Family Violence in Canada: a statistical profile.. Ottowa: Canadian Centre for Justice Statistics; 2005.

(3) Richardson J, Coid J, Petruckevitch A, Chung WS, Moorey S, Feder G. Identifying domestic violence: cross sectional study in primary care. BMJ 2002; 324(7332):274-278.

(4) Donaldson A, Marshall LA. Domestic Abuse Prevalence: Arygll and Clyde DAP study. . Glasgow: West Dunbartonshire Domestic Abuse Partnership; 2005

(5) Feder G. Responding to intimate partner violence: what role for general practice? Br J Gen Pract 2006; 56(525):243-244.

(6) Walby S. The cost of domestic violence. London: Department of Transport and Industry; 2004.

(7) Feder GS, Hutson M, Ramsay J, Taket AR. Women exposed to intimate partner violence: expectations and experiences when they encounter health care professionals: a meta-analysis of qualitative studies. Arch Intern Med 2006; 166(1):22-37.

(8) Dutton MA. Empowering and healing the battered woman: a model for assessment and intervention. New York: Springer; 1992.

(9) Herman JL. Trauma and recovery. London: Pandora; 2001.

(10) Campbell JC. Health consequences of intimate partner violence. Lancet 2002; 359(9314):1331-1336.

(11) Coker AL, Davis KE, Arias I, Desai S, Sanderson M, Brandt HM et al. Physical and mental health effects of intimate partner violence for men and women. Am J Prev Med 2002; 23(4):260-268.

(12) Coker AL, Sanderson M, Dong B. Partner violence during pregnancy and risk of adverse pregnancy outcomes. Paediatr Perinat Epidemiol 2004; 18(4):260-269.

(13) Golding JM. Intimate partner violence as a risk factor for mental disorders: a meta-analysis. Journal of Family Violence 1999; 14:99-132.

(14) Coid J, Petruckevitch A, Chung WS, Richardson J, Moorey S, Feder G. Abusive experiences and psychiatric morbidity in women primary care attenders. Br J Psychiatry 2003; 183:332-339.

(15) Bybee DI, Sullivan CM. The process through which an advocacy intervention resulted in positive change for battered women over time. Am J Community Psychol 2002; 30(1):103-132.

(16) Ramsay J, Feder G, Rivas C. *Interventions to reduce violence and promote the physical and psychosocial well-being of women who experience partner abuse: a systematic review.* London:Department of Health; 2006.

(17) Feder GS, Arsene C, Bacchus L, Dunne D, Hague G, Kuntze S et al. How far does screening women for domestic (partner) violence in different health care settings meet the UK National Screening Committee criteria for a screening programme in terms of condition, screening method, and intervention? Southampton: Health Technology Assessment Programme (In press)

(18) Rees A, Agnew-Davies A, Barkham M. Outcomes for women escaping domestic violence. In: *Society for Psychotherapy Research Book of Abstracts: From research to practice;* 37th International meeting; Edinburgh;2006.

(19) Agnew-Davies A. Reframing psychological symptoms in the context of domestic violence. In: *Society for Psychotherapy Research Book of Abstracts: From research to practice;* 37th International meeting; Edinburgh;2006.

(20) Prochaska JO, Diclemente CC. Stages of change in the modification of problem behaviors. Prog Behav Modif 1992; 28:183-218.

(21) Dutton MA. Empowering and healing the battered woman: a model for assessment and intervention. Springer: NY; 1992.

(22) Hoyt MF. Some stories are better than others: doing what works in brief therapy and managed care. New York:Guildford Press; 2000.

(23) Hegarty K, Fracgp, Bush R, Sheehan M. The composite abuse scale: further development and assessment of reliability and validity of a multidimensional partner abuse measure in clinical settings. Violence Vict 2005; 20(5):529-547.

(24) Evans C, Connell J, Barkham M, Margison F, McGrath G, Mellor-Clark J et al. Towards a standardised brief outcome measure: psychometric properties and utility of the CORE-OM. Br J Psychiatry 2002; 180:51-60.

(25) Hegarty K, Fracgp, Bush R, Sheehan M. The composite abuse scale: further development and assessment of reliability and validity of a multidimensional partner abuse measure in clinical settings. Violence Vict 2005; 20(5):529-547.

(26) Spencer L, Ritchie J, Lewis J, Dillon L. Quality in Qualitative Evaluation: A Framework for Assessing Research Evidence. 2003.

(27) Pope C, Ziebland S, Mays N. Analsysing qualitative data. Br Med J 2000; 320:114-116.

1. [↑](#endnote-ref-2)
2. [↑](#endnote-ref-3)
