# Supplementary material for: Psychological advocacy towards healing (PATH): A randomized controlled trial of a psychological intervention in a domestic violence service setting
Source: PLoS One. 2018 Nov 27;13(11):e0205485. doi: 10.1371/journal.pone.0205485 (PMC6258512; doi:10.1371/journal.pone.0205485)
Supplement: S3 Text — (PDF) [file pone.0205485.s008.pdf]

Brierley et al. Trials 2013, 14:221
http://www.trialsjournal.com/content/14/1/221

TRIALS

STUDY PROTOCOL Open Access

Psychological advocacy toward healing (PATH): study protocol for a randomized controlled trial

Gwen Brierley[1], Roxane Agnew-Davies[2], Jayne Bailey[1], Maggie Evans[1], Morgan Fackrell[3], Giulia Ferrari[1], Sandra Hollinghurst[1], Louise Howard[4], Emma Howarth[1], Alice Malpass[1], Carol Metters[5], Tim J Peters[1], Fayeza Saeed[1], Lynnmarie Sardhina[1], Debbie Sharp[1] and Gene S Feder[1*]

Abstract

Background: Domestic violence and abuse (DVA), defined as threatening behavior or abuse by adults who are intimate partners or family members, is a key public health and clinical priority. The prevalence of DVA in the United Kingdom and worldwide is high, and its impact on physical and mental health is detrimental and persistent. There is currently little support within healthcare settings for women experiencing DVA. Psychological problems in particular may be difficult to manage outside specialist services, as conventional forms of therapy such as counseling that do not address the violence may be ineffective or even harmful. The aim of this study is to assess the overall effectiveness and cost-effectiveness of a novel psychological intervention tailored specifically for survivors of DVA and delivered by domestic violence advocates based in third-sector organizations.

Methods and study design: This study is an open, pragmatic, parallel group, individually randomized controlled trial. Women ages 16 years and older experiencing domestic violence are being enrolled and randomly allocated to receive usual DVA agency advocacy support (control) or usual DVA agency support plus psychological intervention (intervention). Those in the intervention group will receive eight specialist psychological advocacy (SPA) sessions weekly or fortnightly, with two follow-up sessions, 1 month and then 3 months later. This will be in addition to any advocacy support sessions each woman receives. Women in the control group will receive usual DVA agency support but no additional SPA sessions. The aim is to recruit 250 women to reach the target sample size. The primary outcomes are psychological well-being and depression severity at 1 yr from baseline, as measured by the Clinical Outcomes in Routine Evaluation–Outcome Measure (CORE-OM) and the Patient Health Questionnaire (PHQ-9), respectively. Secondary outcome measures include anxiety, posttraumatic stress, severity and frequency of abuse, quality of life and cost-effectiveness of the intervention. Data from a subsample of women in both groups will contribute to a nested qualitative study with repeat interviews during the year of follow-up.

Discussion: This study will contribute to the evidence base for management of the psychological needs of women experiencing DVA. The findings will have important implications for healthcare commissioners and providers, as well as third sector specialist DVA agencies providing services to this client group.

Trial registration: ISRCTN58561170

* Correspondence: gene.feder@bristol.ac.uk
[1]Centre for Academic Primary Care, School of Social and Community Medicine, University of Bristol, Bristol, UK
Full list of author information is available at the end of the article

BioMed Central

© 2013 Brierley et al.; licensee BioMed Central Ltd. This is an Open Access article distributed under the terms of the Creative Commons Attribution License (http://creativecommons.org/licenses/by/2.0), which permits unrestricted use, distribution, and reproduction in any medium, provided the original work is properly cited.

Background

Domestic violence and abuse (DVA) is threatening behavior, violence or abuse (psychological, physical, sexual, or financial), characterized by coercive control, perpetrated by adults who are or have been intimate partners or family members. DVA is common; the 2009/10 British Crime Survey [1] reported that 29% of women had experienced abuse from a partner, ex-partner or family member at some time in their lives. The World Health Organization multicountry study (15 sites in 12 countries) [2] reported estimated lifetime prevalence rates of physical or sexual partner violence, or both, that ranged from 15% to 71% worldwide. Women who experience DVA often have poor physical and mental health, thus making DVA a key public health and clinical priority [3]. The cost of DVA to the UK economy, including costs to the government and employers as well as in terms of human suffering, has been estimated to be £16 billion [4].

Over and above damage to physical health [5,6] and reproductive health [7], DVA has long-term detrimental effects on mental health. It is the leading contributor to the global burden of mental health problems among women of reproductive age [8]. The impact of DVA has psychological parallels with the trauma of being taken hostage and subjected to torture [9,10]. Golding's landmark meta-analysis [11] of 41 studies, mostly North American, measuring the relationship between domestic violence and mental illness, reported increased risks for a range of conditions including depression, posttraumatic stress disorder (PTSD), substance use and suicidality. Our UK-based study of women with a lifetime experience of DVA attending general practices in East London [12] found odds ratios greater than 3 for depression, anxiety and PTSD and greater than 2 for suicide attempts, use of illegal drugs and alcohol abuse.

There is limited evidence for the effectiveness of psychological interventions for victims of DVA. Access to psychological support within the National Health Service (NHS) in the United Kingdom is often difficult [13], and standard psychological interventions such as counseling and cognitive-behavior therapy (CBT) that are not adapted to the specific needs of this vulnerable group have often failed to meet their needs. Service users have found it unhelpful when interventions do not recognize trauma, make the abuser invisible by focusing exclusively on the mental health of the victim (implicitly or explicitly), blame the victim for the abuse or her reaction, offer medication rather than counseling and assign a psychiatric diagnosis that negatively affects care or contact proceedings. In contrast, women identify interventions as helpful when they are encouraged to name domestic violence, are directly asked about their experiences of abuse, are helped with safety planning or parenting and are offered support to recover from their experiences [14]. Psychological interventions may not directly address the violence, and couples or family therapy in which the victim and perpetrator are treated conjointly are potentially dangerous [15,16]. A systematic review [17] found very few studies that examined the effectiveness of different interventions in reducing psychological distress or improving mental health outcomes of women who experience DVA. Modest improvements have been shown in two randomized controlled trials [18,19] using an individual cognitive therapy–based intervention for the treatment of posttraumatic stress in DVA survivors who are no longer in abusive relationships. However, the results of these studies cannot be extrapolated to women with other mental health conditions or to women still in abusive relationships.

A novel psychological intervention specifically tailored for women experiencing DVA, Psychological Advocacy Towards Healing (PATH), has been developed by Agnew-Davies and colleagues [20]. The PATH model draws from different concepts and technical strategies within CBT, experiential, dynamic, psychoeducational and feminist theories. This model has been piloted with a sample of 106 women in refuge settings [20,21] and has been shown to lead to a reduction in women's psychological distress as assessed by the clinical outcomes measured using a routine evaluation outcome measure, the Clinical Outcomes in Routine Evaluation–Outcome Measure (CORE-OM). In that pilot study, using a before-and-after design, the researchers found there was a reduction in mean total CORE score from 1.72 to 1.34 (conventional clinical cut-off of 1.29).

Women with a recent history of DVA have mental health needs that are not being met in primary care or mental health services. The United Kingdom has a network of DVA advocacy and support services, most of which are affiliated with the Women's Aid Federation (http://www.womensaid.org.uk/). Advocates engage with individual clients who are being or have been abused, aiming to empower them and linking them with community services. The core activities of advocacy are provision of legal, housing and financial advice; facilitating access to and use of community resources such as refuges or shelters, emergency housing; provision of safety planning advice; and provision of ongoing support. The duration and intensity of advocacy varies within and between agencies. Generally, advocates do not have a background or training in psychological therapies and do not provide counseling or other therapies. Although advocacy may reduce recurrence of violence, its effect on mental health and quality of life is uncertain [22].

In this study, we are training DVA advocates in the PATH model to enable them to deliver a specialist trauma-focused intervention to the women accessing their services. This trial will evaluate the model's overall

effectiveness and cost-effectiveness. The primary objective of the PATH trial is to determine the extent to which women experiencing DVA who are referred or have self-referred to a specialist DVA agency will have improved quality of life and mental health outcomes if they receive a psychological intervention alongside DVA agency support compared with DVA agency support alone. Secondary objectives are to compare the cost-effectiveness of the two interventions and to investigate differences in the severity and frequency of abuse. A nested qualitative study to explore client perceptions of the intervention to inform interpretation of the results will also be conducted.

Methods

Design

This is an open, pragmatic, two-parallel-group, individually randomized controlled trial. Women seeking help from specialist DVA agencies are randomized at a 1:1 ratio to usual DVA agency support or usual DVA agency support plus additional psychological support from a specially trained advocate or support worker. Two hundred fifty women are being recruited into the study at two DVA agencies, both in the United Kingdom. Participants are enrolled in the study for 1 yr, during which time they will all have access to usual support from the DVA agency. The primary outcome is assessed at 1 yr from randomization.

Participants

Participants are eligible for inclusion if they are experiencing domestic violence or abuse which has led them to seek support from one of the recruiting sites and are age 16 years or older. Women are excluded if they (1) have a psychotic illness, (2) have a severe drug or alcohol problem, (3) are unable to read English or (4) are currently attending counseling, CBT or other psychological treatments in either primary care or specialist psychiatric services.

Recruitment is taking place at two sites: Bristol Next Link (http://www.nextlinkhousing.co.uk/) and Cardiff Women's Aid (http://www.cardiffwomensaid.org.uk/). All women who present to the DVA agencies are assessed for eligibility and invited to consider participation as part of the service's routine intake procedures. Women who are potentially interested in participating are then referred to a female researcher who meets with them face-to-face at a safe and convenient location.

All women referred to the study are provided with a written patient information sheet and have the opportunity to discuss the study with the researcher. Informed consent is obtained from each participant in writing. After the woman consents to participate in the study, the researcher works with her to develop an individualized schedule which includes safety information and the details of friends, family or associates (locators) who will be able to help the researcher to contact the woman if contact is lost. The participant is then asked to complete a baseline questionnaire. On completion, the researcher randomizes the participant by using a remote, independent, automated telephone randomization service provided by the Bristol Randomized Trials Collaboration (http://www.bristol.ac.uk/social-community-medicine/centres/brtc/). Randomization is stratified by whether women receive support from the safe house team or from the resettlement team at Next Link and the refuge team or tenant support team at Cardiff Women's Aid. The randomization program applies stratification with random blocking. Within each stratum, participants are assigned to the treatment group (T) or control group (C) in randomly chosen blocks of two, four or six. Within each block, the order of assignment is also random, so that in a group of four, for example, the permutation TCTC would be equally as likely as TTCC. For each completed block, the allocation ratio is 1:1, so that the number of Ts and Cs will be equal. Allocation is concealed from the researcher until the moment of randomization.

Over the course of the study, women are requested to complete three further questionnaires, at 4, 8 and 12 mo after randomization. Questionnaires are either hand-delivered or sent to the women, depending on the stated preference. Women receive a £10 shopping token after completing the baseline, 4- and 8-month questionnaires and £20 for completion of the 12-months questionnaire. To retain women in the study, researchers make mid-point contacts with women between follow-up points. When researchers are unable to contact a woman on four consecutive occasions, they begin to work through the locator contacts given by the women at the point of recruitment into the study. When a woman cannot be located, researchers continue to use the woman's last known details. Women are prompted to return questionnaires at 2, 4 and 6 wk after the questionnaires are sent or delivered. Failure to return a questionnaire by week 7 prompts the researcher to begin working through the women's locator contacts. Researchers continue to prompt women (or their locators) for the return of questionnaire until week 12 after posting. The researcher will call the woman by telephone and, if no answer is received, leave a voice message (unless it has been explicitly stated that it is not safe to do so). After two failed telephone contact attempts, the researcher will send a text message to the woman's last known mobile telephone number. If there is still no response, the researcher sends a handwritten card to the woman's last known address. Failure to return a questionnaire by week 12 (after it was sent or delivered) results in the questionnaire's being noted as missing. Researchers will continue to issue the next

questionnaire in the series even if the previous questionnaire was not returned.

Intervention

Women randomized to receive psychological support in addition to usual DVA agency support will be assigned a specialist psychological advocate (SPA) who has gone through a 25-day manualized training program from a clinical psychologist with expertise in DVA (RAD). The training addresses the psychological impacts of DVA on women and develops therapeutic skills specifically tailored for this client group. SPAs are trained to work with women with common presenting problems within a single-session model [23] using a session structure (finding a focus for work, obtaining a commitment to work, working on a specific topic, evaluation of progress and homework) based on the work of Daldrup and colleagues [24]. Topics covered include posttraumatic stress, depression, anxiety, low self-esteem, unresolved anger and managing loss. SPAs are also provided with handouts and self-help resources that can be used with their clients. Supervision of the SPAs is provided by the clinical psychologist, who listens to a sample of recorded SPA sessions and provides feedback through regular telephone or email contact and periodic face-to-face meetings.

Once assigned a SPA, the participant will attend eight SPA sessions, meeting either weekly or fortnightly during the course of 2 to 3 months, with a further two booster sessions, 1 and then 3 months later. During SPA sessions, the advocates will provide time-limited interventions using a variety of primarily CBT psychological techniques, focusing within any one session on a specific presenting problem, such as hyperarousal, sleeping difficulties or parenting problems. The advocate will aim to empower the client to apply therapeutic strategies (such as relaxation, challenging thoughts or goal-setting) to promote recovery from each problem. Each series of eight sessions varies according to the client's presenting problems, but typically includes discussions about the impact of domestic abuse on mental health and common reactions, typically including posttraumatic stress and low self-esteem. The client selects a specific topic for each session, and the SPA will use written resources to guide identification of signs (symptoms), the association with experiences of abuse and strategies to overcome them within that session, and through homework tasks to be completed between sessions. Each of the eight sessions can have different content for any therapeutic dyad, although SPAs will focus on building the alliance and safety in the first session and address endings and closure in the eighth session. The follow-up sessions 1 and 3 months after the eighth session are focused on consolidating gains.

In addition, the woman will receive the usual support or advocacy provided by the DVA agencies. This support can involve assistance with a variety of health and social issues, including housing problems, budgeting and debt, and legal proceedings. There is evidence that advocacy on its own can reduce physical abuse and possibly improve quality-of-life outcomes for women who have experienced DVA. Importantly, usual advocacy also provides a space for women to share their experiences and talk about their feelings in a safe and nonjudgmental environment.

Control participants will have access to the usual DVA agency support and advocacy as described above. They will not receive SPA sessions, and their support worker advocate will not have received specialist training in psychological methods. The length of time a woman can be engaged with a DVA agency varies, depending on their needs and the service's policies. Support can range from a one-off session with onward referral to regular meetings over a period of months.

We are not using an attention control group, as this trial is designed (and funded) explicitly as a pragmatic (effectiveness), not explanatory (efficacy), trial, building on previous trial evidence for the effectiveness of psychological and advocacy interventions. Therefore, it is legitimate to have a treatment-as-usual rather than an attention control group. We will collect data on contact time between advocates and participants in both arms and will use that in our interpretation of the findings.

Primary outcomes

This study has two primary outcomes, both collected at 1 yr after randomization. The first is the Clinical Outcomes in Routine Evaluation–Outcome Measure (CORE-OM) score [25]. CORE-OM is a standardized, validated global measure of psychological distress based on a self-completed 34-item questionnaire, with established sensitivity to change, good test-retest reliability and UK normative data. It was designed to assess efficacy and effectiveness across multiple disciplines offering psychological therapies [26]. The second is the Patient Health Questionnaire (PHQ-9) score [27], which is a widely used, self-completed measure of depression [27] with extensive validation in diverse populations with sensitivity to change, leading to its adoption across a wide range of trials internationally.

Secondary outcomes

Some further validated mental health measures are being used: the Generalized Anxiety Disorder 7 scale (GAD-7) [28], the Posttraumatic Stress Disorder CheckList developed by Weathers *et al.* [29], the Composite Abuse Scale to measure type and severity of abuse [30], the Short Form-12 Health Survey to measure quality of life [31]

and the EuroQol EQ-5D to measure health state utility [32]. All secondary outcomes will be measured at 4, 8 and 12 mo by questionnaire. Data derived from the PHQ-9 and CORE-OM questionnaires at 4 and 8 months will also be treated as secondary outcomes and will be used for imputation in the absence of 12-month outcome.

For the economic evaluation, data will be collected on the women's use of health, social and criminal justice services. We will also collect data on expenses incurred by the women for attending appointments, such as travel and child-care costs. Resource use by children of participants will also be measured, such as appointments with youth offender teams and school attendance, as many of the effects of DVA on children and young people manifest as psychological, social, educational and/or behavioral difficulties [33-35]. These data will be collected by questionnaire at the same time points as the above outcome measures.

Other data

Other variables collected in the participant questionnaires are age, number of children at home, ethnicity, income, occupation and relationship to perpetrator. The following process data are recorded by the domestic violence advocates working with the participant: number of DVA agency sessions the participant attends; the main focus of each session; and advocates' contact time with participants, number of women entering the DVA service who do not meet the eligibility criteria or who are not interested in participating. We will also collect data on adverse events self-reported by the participant (verbally and/or by questionnaire) or elicited by the DVA advocate following contact with her.

Sample size

Using pilot data, the sample size is based on being able to detect a difference of 0.5 in the CORE-OM score, a "reliable change index" [25]. This will be the difference in psychological distress between intervention and control groups as measured by the CORE-OM questionnaire 12 months after each patient is randomly assigned. A sample size of 200 participants in total gives a power of 96% to detect a difference of 0.5 on the CORE-OM, corresponding to an effect size of 0.5 and 81% power to detect an effect size of 0.4. Assuming an attrition of 20%, we will need to recruit 250 women.

An effect size of 0.4 to 0.5 is consistent with effect sizes detected in studies of psychological interventions using the CORE-OM as an outcome measure [36] and is similar to that of CBT interventions on the PHQ-9 and other depression outcome measures [37]. An effect size in the 0.4 to 0.5 range is also comparable to the outcome of trials of psychological interventions for women who have experienced domestic violence (see Appendix 7.5 in our systematic review of interventions [38]). For example, Kubany and colleagues' two trials of CBT for women diagnosed with PTSD following intimate partner violence reported effect sizes of 0.9 [19] and 0.33 [18], respectively, for PTSD and 0.4 and 0.25, respectively, for depression outcomes. Labrador *et al.* [39] reported effect sizes of 1.23 and 1.77 for PTSD and depression outcomes, respectively, and Reed and Enright [40] reported effect sizes of 2.33 and 1.55 for PTSD and depression, respectively.

Nested qualitative study

A sample of approximately 30 women across both trial groups from both recruiting sites will be invited to take part in a series of three interviews spaced across their year of participation. The aim of these interviews will be to explore women's assessment of their mental well-being and quality of life in their own words, as well as the overall acceptability and perceived effectiveness of the services they received. Topic guides include questions that explore which of the therapeutic strategies (such as exercises to resolve anger, challenging negative thoughts or goal setting) were most useful to the women and why. Interviews are face-to-face and will last up to one hour. With participants' consent, interviews will be recorded and transcribed verbatim. Purposive sampling based on data collected in the baseline questionnaires will ensure a diverse group of informants in terms of age, currency of abuse, and mental health status.

Statistical analysis

Trial outcomes will be analyzed and reported in accordance with Consolidated Standards of Reporting Trials (CONSORT) guidelines. Baseline demographic data such as gender, age, duration of abuse and relationship to perpetrator will be summarized and descriptive summary statistics will be provided to check for imbalances between the two trial groups. For variables with continuous measures, we will report the mean and standard deviation, and for categorical data, we will report frequencies and percentages.

Because the allocation will be stratified by site (Bristol vs Cardiff) and type of setting where the treatment is administered (safe house vs within community), the comparative analyses will adjust for these two factors. These will involve normal and logistic regression analyses for continuous and binary outcomes, respectively, and also, where appropriate, will adjust for the baseline scores of the outcome variables. The primary comparative analysis will compare continuous CORE-OM scores between the two groups as randomized using an intention-to-treat regression model. Secondary analysis will adjust for any factors exhibiting substantial baseline imbalance; further secondary analyses will include prespecified

subgroup analyses according to the two stratification variables and age, which will be investigated by introducing the relevant interaction with treatment group into the regression model [41]. It is possible that the treatment effect will differ across both sites and settings because of demographic differences between Cardiff and Bristol and differences in community and safe house or refuge settings. It is also likely that treatment impact will differ between age groups, partly in connection with baseline exposure to violence, which tends to be higher in the younger strata of the population [1,42]. The subgroup analysis by age will use the median as a threshold to separate younger from older women in the sample as well as use age as a continuous variable in two separate specifications. Because rapport with the therapist (in this case, the specialist psychological advocate) is a key component of therapy effectiveness [43-45], we will also account for potential differences in impact at the therapist level in generalized mixed models. To assess the stability of any treatment effect, we will fit a mixed model for CORE-OM or PHQ-9 scores at 4, 8 and 12 months, adjusted for baseline CORE-OM or PHQ-9, using number of treatment sessions as a fixed categorical effect.

The impact of, and reasons for, missing data will be investigated in sensitivity analyses based on multiple imputation by chained equation models (mice) [46-49]. The imputation model will contain all the variables predictive of missingness and of the outcome, as well as those in the hypothesized model [48]. The number of imputations will be determined on the basis of the percentage of data that will be missing, as well as on the precision of the estimate [48]. A random coefficient mixed effects model will also be considered in comparing groups in terms of CORE-OM and PHQ-9 scores. A mixed (fixed and random) effects model will allow us to account for the distribution of errors, compared with fixed effects models [50] and to deal with missing data were these not to be missing at random [51].

We will calculate complier-average causal effect (CACE) using instrumental variables techniques to establish a stronger causal link between assignment to treatment and impact in the absence of full adherence to the treatment [46,52,53]. In particular, it is possible that different individuals will complete treatment at a different pace than that envisaged by the trial team, and these models will take this heterogeneity into account. To calculate CACE estimates, adherence will be represented primarily by the number of treatment sessions attended. Where a binary version is required, we will in the first instance consider completion of at least four sessions at 12 months from randomization as reflecting adequate adherence.

Economic analysis

Although there is literature on the economic impact of DVA, to date there has been little economic analysis of interventions aimed at improving outcomes for domestic violence survivors [54]. The aim of the economic evaluation is to compare the extra cost of providing the PATH intervention with the extra benefits gained to allow judgment about its cost-effectiveness. This process involves detailed individual-level costing of caring for women who are victims of DVA. The cost of caring for women offered the PATH intervention (that is, sessions with the SPAs) will be compared with the cost of domestic violence advocacy alone. The analysis will consider costs associated with the delivery of care from the perspectives of the NHS and personal social services (PSSs), the public sector and society in general. Resource use will be measured from the point of randomization until final follow-up 12 mo later. Data will be collected by means of a questionnaire at 4, 8 and 12 mo, and the analysis will include all resources used by the women unless clearly unrelated to domestic violence, including relevant resources used by or on behalf of children younger than age 17 years in their care.

We will report the direct costs of the intervention. Because the training curriculum was developed prior to the intervention [20,21], start-up costs pertain only to training of the SPAs. Delivering the intervention requires SPA time, clinical supervision and management by the host domestic violence agency. We will quantify time with the use of timesheets and the original budgets. This time will be valued at advocates' and trainers' salaries and management costs paid to the host agencies.

Use of NHS and PSS resources will be captured through the questionnaire. These will include primary and community health services, prescribed medication and use of hospital services. Unit costs for the valuation of these resources will be derived using the method described by Curtis [55], Department of Health reference costs and the British National Formulary. Community and social care services (such as family therapists and social workers) provide day-to-day support to DVA victims and their children to help them cope with the trauma and aftermath of domestic abuse. We will measure residence in refuges or safe houses by participants during the 1-yr follow-up period, estimating costs from information given by participating agencies and other refuge providers. Use of legal services as a consequence of DVA will be measured using our questionnaire, and we will derive relevant unit costs from current aggregate estimates [56]. Finally, in line with the societal perspective in this analysis, we will also quantify personal and social costs caused by the exposure to DVA. These will include personal outlay for travel, child care and change in housing circumstances, as well as the use of voluntary sector services and time off from work.

We will present results using a cost consequences framework comparing cost from each perspective with

primary and secondary outcomes [57]. We will use the EQ-5D data from the study questionnaires to construct quality-adjusted life years (QALYs) and present an incremental cost-effectiveness ratio. This will indicate the cost per QALY gained by adding a psychological intervention to advocacy for women experiencing DVA. Costs and outcomes will not be discounted, as the study will be limited to a period of 12 mo. The effect of uncertainty in unit cost estimates or assumptions about resource use will be addressed in sensitivity analyses. Uncertainty in the cost-effectiveness/utility ratios resulting from participant variation in resource use and effectiveness will be captured by estimating confidence intervals around the net benefit statistic and estimating cost-effectiveness acceptability curves.

Qualitative analysis

The aims of the qualitative analysis will be twofold: first to understand the meaning and perceived effectiveness of the intervention in the context of the women's lives, and second to deepen our understanding of the process of identity change for women as they move through some or all of the stages from victim to survivor to thriver [58]. The first analytic strand will provide important insights into contextual and motivational factors for the women in relation to the intervention. This will enable hypotheses to be tested about factors likely to act as barriers or facilitators to the effectiveness of the intervention, for example, practical arrangements for SPA sessions, such as the availability of a safe location or child care or finding suitable times to attend sessions, as well as factors relating to women's expectations or prior experiences in help-seeking and their relationships with provider organizations and individual SPA workers. Comparisons will be made with the help-seeking experiences of women in the control arm of the study. The second analytic strand will be conducted using the perspective of interpretive phenomenological analysis which explores the lived experience of participants [59]. This approach allows for a deeper exploration and interpretation of women's experiences. Insights gained will enable the research team to critique and add to the theoretical development in the field of DVA, most of which has been generated in the United States [60-63].

Discussion

This study is the first randomized controlled trial of an intervention to improve the psychological well-being of women experiencing DVA in a UK setting. The study is a collaboration with third-sector agencies that already provide a valuable service for this client group through provision of specialist support and advocacy. If a psychological program delivered by DVA advocates improves mental health outcomes for women who have experienced DVA, and if this is a cost-effective intervention, the PATH model will be appropriate for commissioning by the NHS.

Study organization

The University of Bristol is the sponsor for this trial. The study is funded through the UK National Institute for Health Research as part of the PROVIDE (Programme of Research On Violence In Diverse domestic Environments) Programme Grant for Applied Research. The Centre for Academic Primary Care at the University of Bristol is coordinating the study and monitoring and verifying the data and will analyze the results. A trial steering committee consisting of an independent chair and two other independent members, along with the lead investigator and other study investigators, will oversee the conduct of the trial. An independent Data Monitoring and Ethics Committee chaired by Harriet MacMillan, a domestic violence trialist, and comprising a statistician, trial methodology specialist and the research and policy officer for England Women's Aid has been set up to review safety and outcome data. Further details about the trial are available at the PROVIDE website (http://www.provide.ac.uk/).

Ethics approval

The study has been approved by the South West Southmead National Research Ethics Service, and site-specific approvals have been received from the appropriate local research ethics committees. The study is being conducted in accordance with the ethical principles in the Declaration of Helsinki and good practice guidelines on the proper conduct of research.

Competing interests

RAD is director of Domestic Violence Training Ltd, a private limited company which includes an aspect of training and consultancy that may potentially benefit from increased business if the trial has a positive outcome. MF is director of Cardiff Women's Aid, and CM is director of Bristol Next Link; both organizations may benefit from commissioning of the PATH program if the trial has a positive outcome. The other authors declare that they have no competing interests.

Authors' contributions

GB managed the research associates, developed standard operating procedures, secured ethics and governance approval, facilitated the trial steering committee and the data management and ethics committee, coordinated adverse event reporting and wrote the first draft of this paper. RAD developed the PATH intervention, contributed to choice of outcome measures and supervised the specialist psychological advocates. JB manages the research associates after the first year of the study, develops further standard operating procedures, facilitates the trial steering committee and the data management and ethics committee and coordinates adverse event reporting. ME conducts the qualitative study. MF manages the specialist psychological advocates at the Cardiff site. GF performed power calculations and drafted the analysis sections of the manuscript. SH designed the economic data collection and analysis. LH contributed to the design of the trial. EH recruits and follows up participants and liaises with Cardiff Women's Aid. AM designed the qualitative study. CM manages the specialist psychological advocates at the Bristol site. TJP designed the trial and supervises the statistical analysis. FS and LS recruited and followed up participants and liaised with Bristol Next Link. DS contributed to the design of the trial. GSF designed the trial and

leads the research team. All the authors contributed to the drafting and review of this paper.

Acknowledgments

We are very grateful to all the women clients of Next Link and Cardiff Women's Aid who will participate in PATH, our trial steering committee (Simon Gilbody and Nicky Stanley), the PROVIDE administrator (Patricia Martens), our Data Monitoring and Ethics Committee (Harriet Macmillan, Jackie Barron, Sandra Eldridge and Michael King) and our patient public involvement group members (Patricia Chipungu, Jackie Evans, Josie Hughes, Maninder Kaur, Maria Rees and Hyat Robbins). This report presents independent research commissioned by the National Institute for Health Research (NIHR) under its Programme Grants for Applied Research scheme (RP-PG-0108-10084). The views expressed in this publication are those of the authors and not necessarily those of the NHS, the NIHR or the Department of Health.

Author details

[1]Centre for Academic Primary Care, School of Social and Community Medicine, University of Bristol, Bristol, UK. [2]Domestic Violence Training Ltd, Surbiton, Surrey, UK. [3]Cardiff Women's Aid, Cardiff, UK. [4]Institute of Psychiatry, King's College London, London, UK. [5]Next Link Bristol, Bristol, UK.

Received: 11 December 2012 Accepted: 26 June 2013
Published: 17 July 2013

References

1. Hall P: **Intimate violence: 2009/10 British crime survey.** In *Homicides, Firearm Offences and Intimate Violence 2009/10*, Home Office Statistical Bulletin (Supplementary Volume 2 to Crime in England and Wales 2009/10). Edited by Smith K. London: Home Office; 2011:68–81. Available at: http://webarchive.nationalarchives.gov.uk/20110218135832/rds.homeoffice.gov.uk/rds/pdfs11/hosb0111.pdf.
2. Garcia-Moreno C, Jansen HAFM, Ellsberg M, Heise L, Watts CH, WHO Multi-country Study on Women's Health and Domestic Violence Against Women Study Team: **Prevalence of intimate partner violence: findings from the WHO multi-country study on women's health and domestic violence.** *Lancet* 2006, **368:**1260–1269.
3. Feder G: **Responding to intimate partner violence: what role for general practice?** *Br J Gen Pract* 2006, **56:**243–244.
4. Walby S: *The Cost of Domestic Violence: Up-date 2009.* Lancaster, UK: Lancaster University/UNESCO Chair in Gender Research; 2009.
5. Campbell JC: **Health consequences of intimate partner violence.** *Lancet* 2002, **359:**1331–1336.
6. Coker AL, Davis KE, Arias I, Desai S, Sanderson M, Brandt HM, Smith PH: **Physical and mental health effects of intimate partner violence for men and women.** *A J Prev Med* 2002, **23:**260–268.
7. Coker AL, Sanderson M, Dong B: **Partner violence during pregnancy and risk of adverse pregnancy outcomes.** *Paediatr Perinat Epidemiol* 2004, **18:**260–269.
8. Vos T, Astbury J, Piers LS, Magnus A, Heenan M, Stanley L, Walker L, Webster K: **Measuring the impact of intimate partner violence on the health of women in Victoria, Australia.** *Bull World Health Organ* 2006, **84:**739–744.
9. Dutton MA: *Empowering and Healing the Battered Woman: A Model for Assessment and Intervention.* New York: Springer; 1992.
10. Herman JL: *Trauma and Recovery: The Aftermath of Violence–From Domestic Abuse to Political Terror.* London: Pandora Press; 2001.
11. Golding JM: **Intimate partner violence as a risk factor for mental disorders: a meta-analysis.** *J Fam Violence* 1999, **14:**99–132.
12. Coid J, Petruckevitch A, Chung WS, Richardson J, Moorey S, Feder G: **Abusive experiences and psychiatric morbidity in women primary care attenders.** *Br J Psychiatry* 2003, **183:**332–341.
13. Lovell K, Richards D: **Multiple Access Points and Levels of Entry (MAPLE): ensuring choice, accessibility and equity for CBT services.** *Behav Cogn Psychother* 2000, **28:**379–391.
14. Humphreys C, Thiara R: **Mental health and domestic violence: 'I call it symptoms of abuse'.** *Br J Soc Work* 2003, **33:**209–226.
15. Galvani S: **Safety in numbers? Tackling domestic abuse in couples and network therapies.** *Drug Alcohol Rev* 2007, **26:**175–181.
16. Tunariu AD: *Bridging to Change: Expanding Relate's Capacity to Provide Work with Clients Who Use Domestic Violence and Abuse and to Meet the Needs of Clients Whose Lives Are Affected by It: A Review of Three Domestic Violence and Abuse Prevention Programmes (DVA-PP).* Lakeside, UK: Relate; 2007.
17. Ramsay J, Rivas C, Feder G: *Interventions to Reduce Violence and Promote the Physical and Psychosocial Well-being of Women Who Experience Partner Violence: A Systematic Review of Controlled Evaluations.* London: Barts and the London School of Medicine and Dentistry, Queen Mary, University of London; 2005. Available at: http://ndvf.org.uk/files/document/897/original.pdf.
18. Kubany ES, Hill EE, Owens JA, Iannce-Spencer C, McCaig MA, Tremayne KJ, Williams PL: **Cognitive trauma therapy for battered women with PTSD (CTT-BW).** *J Consult Clin Psychol* 2004, **72:**3–18.
19. Kubany ES, Hill EE, Owens JA: **Cognitive trauma therapy for battered women with PTSD: preliminary findings.** *J Trauma Stress* 2003, **16:**81–91.
20. Agnew-Davies R: **Reframing psychological symptoms in the context of domestic violence.** In *Society of Psychotherapy Research Book of Abstracts: From Research to Practice.* 37th International Meeting, June 21–24, Edinburgh, UK.
21. Rees A, Agnew-Davies R, Barkham M: **Outcomes for women escaping domestic violence.** In *Society for Psychotherapy Research Book of Abstracts: From Research to Practice.* 37th International Meeting, June 21–24, Edinburgh, UK.
22. Ramsay J, Carter Y, Davidson L, Dunne D, Eldridge S, Feder G, Hegarty K, Rivas C, Taft A, Warburton A: **Advocacy interventions to reduce or eliminate violence and promote the physical and psychosocial well-being of women who experience intimate partner abuse.** *Cochrane Database Syst Rev* 2009, **3:**CD005043.
23. Hoyt MF: *Some Stories Are Better Than Others: Doing What Works in Brief Therapy and Managed Care.* New York: Routledge; 2000.
24. Daldrup RJ, Beutler LE, Engle D, Greenberg LS: *Focused Expressive Psychotherapy: Freeing the Overcontrolled Patient.* London: Guilford Press; 1988.
25. Barkham M, Mellor-Clark J, Connell J, Cahill J: **A core approach to practice-based evidence: a brief history of the origins and applications of the CORE-OM and CORE System.** *Couns Psychother Res* 2006, **6:**3–15.
26. Evans C, Connell J, Barkham M, Margison F, McGrath G, Mellor-Clark J, Audin K: **Towards a standardised brief outcome measure: psychometric properties and utility of the CORE-OM.** *Br J Psychiatry* 2002, **180:**51–60.
27. Spitzer RL, Kroenke K, Williams JBW, and the Patient Health Questionnaire Primary Care Study Group: **Validation and utility of a self-report version of PRIME-MD: the PHQ Primary Care Study.** *JAMA* 1999, **282:**1737–1744.
28. Spitzer RL, Kroenke K, Williams JBW, Löwe B: **A brief measure for assessing generalized anxiety disorder: the GAD-7.** *Arch Intern Med* 2006, **166:**1092–1097.
29. Weathers FW, Litz BT, Huska J, Keane TM: *PTSD CheckList–Civilian Version (PCL-C).* Boston: VA National Center for PTSD–Behavioral Science Division; 1994. Available at: http://www.mirecc.va.gov/docs/visn6/3_PTSD_CheckList_and_Scoring.pdf.
30. Hegarty K, Fracgp, Bush R, Sheehan M: **The composite abuse scale: further development and assessment of reliability and validity of a multidimensional partner abuse measure in clinical settings.** *Violence Vict* 2005, **20:**529–547.
31. Gandek B, Ware JE, Aaronson NK, Apolone G, Bjorner JB, Brazier JE, Bullinger M, Kaasa S, Leplege A, Prieto L, Sullivan M: **Cross-validation of item selection and scoring for the SF-12 health survey in nine countries: results from the IQOLA project: international quality of life assessment.** *J Clin Epidemiol* 1998, **51:**1171–1178.
32. The EuroQol Group: **EuroQol: a new facility for the measurement of health-related quality of life.** *Health Policy* 1990, **16:**199–208.
33. Kitzmann KM, Gaylord NK, Holt AR, Kenny ED: **Child witnesses to domestic violence: a meta-analytic review.** *J Consult Clin Psychol* 2003, **71:**339–352.
34. Chan YC, Yeung JWK: **Children living with violence within the family and its sequel: a meta-analysis from 1995–2006.** *Aggress Violent Behav* 2009, **14:**313–322.
35. Evans SE, Davies C, DiLillo D: **Exposure to domestic violence: a meta-analysis of child and adolescent outcomes.** *Aggress Violent Behav* 2008, **13:**131–140.
36. Barkham M, Stiles WB, Connell J, Twigg E, Leach C, Lucock M, Mellor-Clark J, Bower P, King M, Shapiro DA, Hardy GE, Greenberg L, Angus L: **Effects of psychological therapies in randomized trials and practice-based studies.** *Br J Clin Psychol* 2008, **47:**397–415.

37. Butler AC, Chapman JE, Forman EM, Beck AT: **The empirical status of cognitive-behavioral therapy: a review of meta-analyses.** *Clin Psychol Rev* 2006, **26:**17–31.
38. Feder G, Ramsay J, Dunne D, Rose M, Arsene C, Norman R, Kuntze S, Spencer A, Bacchus L, Hague G, Warburton A, Taket A: **How far does screening women for domestic (partner) violence in different health-care settings meet the criteria for a screening programme? Systematic reviews of nine UK National Screening Committee criteria.** *Health Technol Assess* 2009, **13:**iii–iv, xi–xiii, 137–147.
39. Labrador FJ, Fernandez-Velasco MD, Rincon PP: **Efficacy of a brief and individual participation program by posttraumatic stress disorder in female victims of domestic violence.** *Int J Clin Health Psychol* 2006, **6:**527–547.
40. Reed GL, Enright RD: **The effects of forgiveness therapy on depression, anxiety, and posttraumatic stress for women after spousal emotional abuse.** *J Consult Clin Psychol* 2006, **74:**920–929.
41. Sun X, Briel M, Busse JW, You JJ, Akl EA, Mejza F, Bala MM, Bassler D, Mertz D, Diaz-Granados N, Vandvik PO, Malaga G, Srinathan SK, Dahm P, Johnston BC, Alonso-Coello P, Hassouneh B, Walter SD, Heels-Ansdell D, Bhatnagar N, Altman DG, Guyatt GH: **Credibility of claims of subgroup effects in randomised controlled trials: systematic review.** *BMJ* 2012, **344:**e1553.
42. Hester M, Donovan C, Fahmy E: **Feminist epistemology and the politics of method: surveying same sex domestic violence.** *Int J Res Methods* 2010, **13:**251–263.
43. Roberts C, Roberts SA: **Design and analysis of clinical trials with clustering effects due to treatment.** *Clinical Trials* 2005, **2:**152–162.
44. Stiles WB, Agnew-Davies R, Barkham M, Culverwell A, Goldfried MR, Halstead J, Hardy GE, Raue PJ, Rees A, Shapiro DA: **Convergent validity of the Agnew relationship measure and the working alliance inventory.** *Psychol Assess* 2002, **14:**209–220.
45. Agnew-Davies R: **Learning from research into the counselling relationship.** In *Professional Skills for Counsellors: Understanding the Counselling Relationship.* Edited by Feltham C. London: Sage Publications Ltd; 1999:200–237.
46. Thomas LJ, Abel A, Ridgway N, Peters T, Kessler D, Hollinghurst S, Turner K, Garland A, Jerrom B, Morrison J, Williams C, Campbell J, Kuyken W, Lewis G, Wiles N: **Cognitive behavioural therapy as an adjunct to pharmacotherapy for treatment resistant depression in primary care: the CoBalT randomised controlled trial protocol.** *Contemp Clin Trials* 2012, **33:**312–319.
47. Thomas L, Mulligan J, Mason V, Tallon D, Wiles N, Cowen P, Nutt D, O'Donovan M, Sharp D, Peters T, Lewis G: **GENetic and clinical Predictors Of treatment response in Depression: the GenPod randomised trial protocol.** *Trials* 2008, **9:**29.
48. Spratt M, Carpenter J, Sterne JA, Carlin JB, Heron J, Henderson J, Tilling K: **Strategies for multiple imputation in longitudinal studies.** *Am J Epidemiol* 2010, **172:**478–487.
49. van Buuren S, Boshuizen HC, Knook DL: **Multiple imputation of missing blood pressure covariates in survival analysis.** *Stat Med* 1999, **18:**681–694.
50. Rapp SR, Espeland MA, Shumaker SA, Henderson VW, Brunner RL, Manson JE, Gass ML, Stefanick ML, Lane DS, Hays J, Johnson KC, Coker LH, Dailey M, Bowen D, WHIMS Investigators: **Effect of estrogen plus progestin on global cognitive function in postmenopausal women: the women's health initiative memory study: a randomized controlled trial.** *JAMA* 2003, **289:**2663–2672.
51. Laird NM, Ware JH: **Random-effects models for longitudinal data.** *Biometrics* 1982, **38:**963–974.
52. Greenland S: **Principles of multilevel modelling.** *Int J Epidemiol* 2000, **29:**158–167.
53. Lawlor DA, Jago R, Noble SM, Chittleborough CR, Campbell R, Mytton J, Howe LD, Peters TJ, Kipping RR: **The active for Life Year 5 (AFLY5) school based cluster randomised controlled trial: study protocol for a randomized controlled trial.** *Trials* 2011, **12:**181.
54. Gold L, Norman R, Devine A, Feder G, Taft AJ, Hegarty KL: **Cost effectiveness of health care interventions to address intimate partner violence: what do we know and what else should we look for?** *Violence Against Women* 2011, **17:**389–403.
55. Curtis L: *Unit Costs of Health & Social Care 2011.* Kent, UK: Personal Social Services Research Unit, University of Kent; 2011. Available at: http://www.pssru.ac.uk/archive/pdf/uc/uc2011/uc2011.pdf.
56. Walby S: *The Cost of Domestic Violence.* London: Women and Equality Unit, Department of Trade and Industry; 2004. Available at: http://www.devon.gov.uk/de/text/cost_of_dv_report_sept04.pdf.
57. Hollinghurst S, Peters TJ, Kaur S, Wiles N, Lewis G, Kessler D: **Cost-effectiveness of therapist-delivered online cognitive-behavioural therapy for depression: randomised controlled trial.** *Br J Psychiatry* 2010, **197:**297–304.
58. Wozniak DF, Allen KN: **Ritual and performance in domestic violence healing: from survivor to thriver through rites of passage.** *Cult Med Psychiatry* 2012, **36:**80–101.
59. Smith JA, Flowers P, Larkin M: *Interpretative Phenomenological Analysis: Theory, Method and Research.* London: Sage Publications Ltd; 2009.
60. Zosky D: **A matter of life and death: the voices of domestic violence survivors.** *Affilia* 2011, **26:**201–212.
61. Chang JC, Dado D, Ashton S, Hawker L, Cluss PA, Buranosky R, Scholle SH: **Understanding behavior change for women experiencing intimate partner violence: mapping the ups and downs using the stages of change.** *Patient Educ Couns* 2006, **62:**330–339.
62. Sleutel MR: **Women's experiences of abuse: a review of qualitative research.** *Issues Ment Health Nurs* 1998, **19:**525–539.
63. Liang B, Goodman L, Tummala-Narra P, Weintraub S: **A theoretical framework for understanding help-seeking processes among survivors of intimate partner violence.** *Am J Community Psychol* 2005, **36:**71–84.

doi:10.1186/1745-6215-14-221
Cite this article as: Brierley *et al.*: Psychological advocacy toward healing (PATH): study protocol for a randomized controlled trial. *Trials* 2013 **14**:221.

Submit your next manuscript to BioMed Central and take full advantage of:

- **Convenient online submission**
- **Thorough peer review**
- **No space constraints or color figure charges**
- **Immediate publication on acceptance**
- **Inclusion in PubMed, CAS, Scopus and Google Scholar**
- **Research which is freely available for redistribution**

Submit your manuscript at
www.biomedcentral.com/submit

BioMed Central
